# Influence of breast cancer risk factors and intramammary biotransformation on estrogen homeostasis in the human breast

**DOI:** 10.1007/s00204-020-02807-1

**Published:** 2020-06-22

**Authors:** Daniela Pemp, Leo N. Geppert, Claudia Wigmann, Carolin Kleider, René Hauptstein, Katja Schmalbach, Katja Ickstadt, Harald L. Esch, Leane Lehmann

**Affiliations:** 1 Institute of Pharmacy and Food Chemistry, University of Würzburg, Am Hubland, 97074 Würzburg, Germany; 2 Mathematical Statistics with Applications in Biometrics, TU Dortmund University, Vogelpothsweg 87, 44221 Dortmund, Germany

**Keywords:** Estrogens, Human breast, Multiple linear regression

## Abstract

Understanding intramammary estrogen homeostasis constitutes the basis of understanding the role of lifestyle factors in breast cancer etiology. Thus, the aim of the present study was to identify variables influencing levels of the estrogens present in normal breast glandular and adipose tissues (GLT and ADT, i.e., 17β-estradiol, estrone, estrone-3-sulfate, and 2-methoxy-estrone) by multiple linear regression models. Explanatory variables (exVARs) considered were (a) levels of metabolic precursors as well as levels of transcripts encoding proteins involved in estrogen (biotrans)formation, (b) data on breast cancer risk factors (i.e., body mass index, BMI, intake of estrogen-active drugs, and smoking) collected by questionnaire, and (c) tissue characteristics (i.e., mass percentage of oil, oil%, and lobule type of the GLT). Levels of estrogens in GLT and ADT were influenced by both extramammary production (menopausal status, intake of estrogen-active drugs, and BMI) thus showing that variables known to affect levels of circulating estrogens influence estrogen levels in breast tissues as well for the first time. Moreover, intratissue (biotrans)formation (by aromatase, hydroxysteroid-17beta-dehydrogenase 2, and beta-glucuronidase) influenced intratissue estrogen levels, as well. Distinct differences were observed between the exVARs exhibiting significant influence on (a) levels of specific estrogens and (b) the same dependent variables in GLT and ADT. Since oil% and lobule type of GLT influenced levels of some estrogens, these variables may be included in tissue characterization to prevent sample bias. In conclusion, evidence for the intracrine activity of the human breast supports biotransformation-based strategies for breast cancer prevention. The susceptibility of estrogen homeostasis to systemic and tissue-specific modulation renders both beneficial and adverse effects of further variables associated with lifestyle and the environment possible.

**Electronic supplementary material:**

The online version of this article (10.1007/s00204-020-02807-1) contains supplementary material, which is available to authorized users.

## Introduction

Breast cancer is the most common cancer in women worldwide. Its development is associated with increased levels of circulating estrogens, e.g., 17β-estradiol (E2), estrone (E1), and other endogenous steroids in pre- and postmenopausal women (Endogenous Hormones Breast Cancer Collaborative Group 2002, 2013) over a prolonged period of time. Based on these associations as well as an abundance of experiments in vitro and in animal models, the current understanding of the molecular etiology of breast cancer hypothesizes biotransformation of E2/E1 within the breast tissue to catechols and subsequent oxidation to mutagenic quinones possibly initiating tumor formation. Tumor promotion is then favored by estrogen receptor (ESR)-mediated stimulation of proliferation of the initiated cells (Yager and Davidson 2006). Thus, both tumor initiation and progression would depend predominately on intramammary levels of reactive products of estrogen biotransformation, whereas tumor promotion would depend predominately on levels of E2.

Consequently, commonly accepted risk factors such as early menarche and/or late menopause, late age at first pregnancy, small number of pregnancies, and short or no periods of breastfeeding (Colditz and Bohlke 2014) are supposed to increase the duration or extent of the local exposure of the mammary gland to E2 (biotransformation products) by increasing their systemic production. Concurrently, current risk reduction strategies involve the chemical modulation of ESR as well as systemic inhibition of aromatase or salpingo-oophorectomy (Advani and Moreno-Aspitia 2014) aimed to reduce levels of circulating estrogens. Recently, also modifiable risk factors associated with lifestyle such as (postmenopausal) obesity, alcohol consumption (Colditz and Bohlke 2014), smoking (Gaudet et al. 2017; Jones et al. 2017; Gram et al. 2019), and intake of estrogen-active drugs (EADs) for oral contraception (Grosse et al. 2009) or hormone replacement therapy (Collaborative Group on Hormonal Factors in Breast Cancer 2019) have been associated with both increased breast cancer risk and higher levels of circulating E2 and E1 (Endogenous Hormones Breast Cancer Collaborative Group 2003, 2011, 2013), suggesting that these risk factors also act by affecting intramammary levels of E2/E1 (biotransformation products).

Given the wide range of enzymes present in breast glandular (GLT) and adipose tissues (ADT; Pemp et al. 2019a), additional (biotrans)formation of estrogens within the breast tissue can reasonably be assumed (Labrie 2015; Mueller et al. 2015; Hilborn et al. 2017; Pemp et al. 2019a). Consequently, breast cancer risk factors may also influence tissue levels of E2 and its biotransformation products by affecting estrogen homeostasis in the breast. Thus, to better understand how estrogen homeostasis may affect initiation and promotion of breast cancer, insight into the influence of breast cancer risk factors on both levels of estrogen and estrogen biotransformation in women without breast cancer is needed.

However, only two studies in women without breast cancer have been published in this regard of which only one has performed statistical analyses (Online Resource 1, Savolainen-Peltonen et al. 2014). Even considering those analyzing non-tumor tissue of women with breast cancer and investigating the association of risk factors with tissue levels of estrogens, most studies did not provide information on parameters statistically compared and whether or not all positive/negative correlations were reported (Online Resource 1). Surprisingly, none of the available studies included reproductive history of the participating women in their statistical analyses, or collected data on smoking or analyzed biotransformation products of E2 other than E1 by recommended methods of specific analysis (Online Resource 1).

**Fig. 1:**
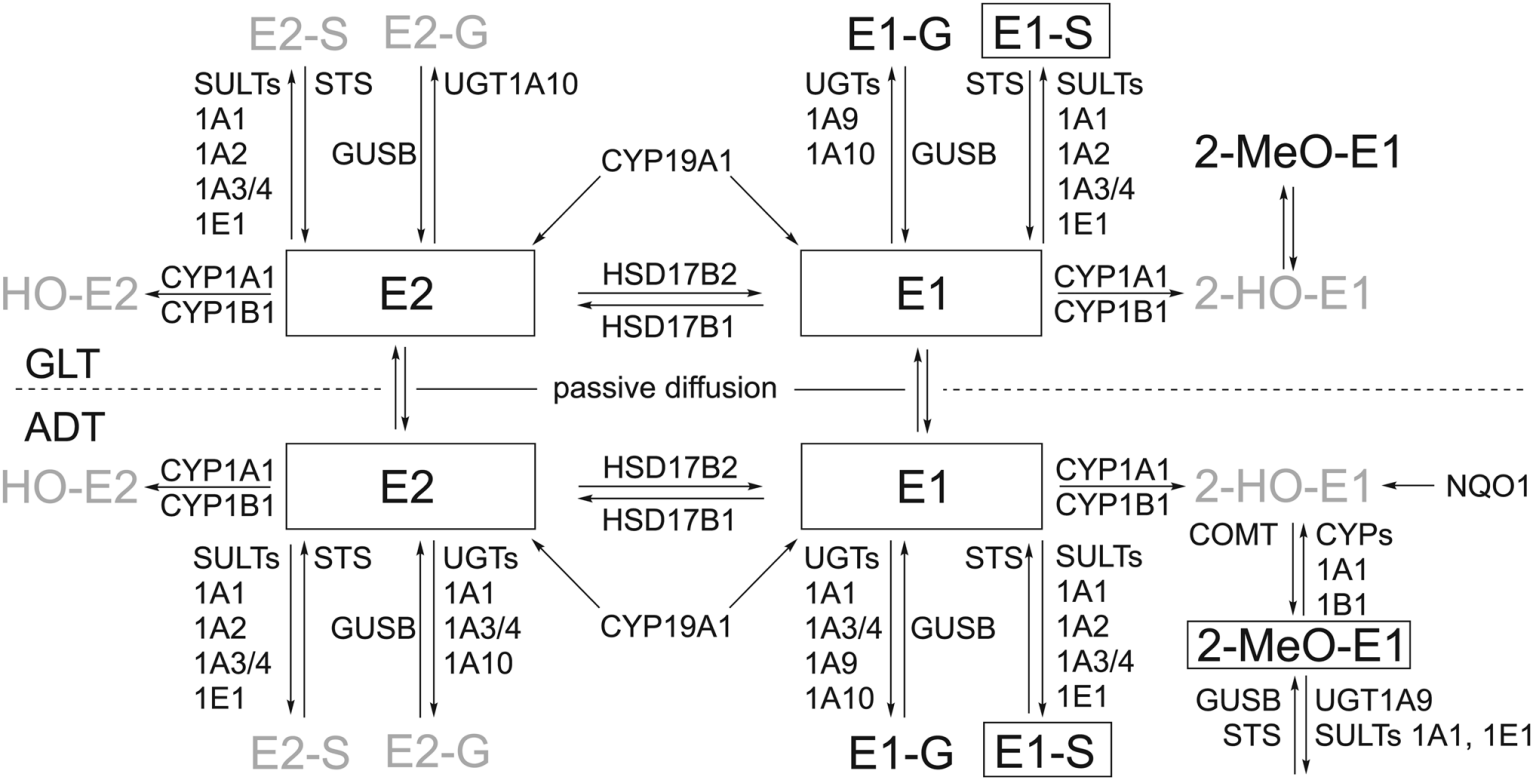
Current knowledge on (biotrans)formation of E2 in breast GLT and ADT of women without breast cancer based on recently published information on quantitative estrogen profiles and levels of transcripts encoding enzymes involved in (biotrans)formation of E2 in human breast tissues (Pemp et al 2019a). Intramammary tissue levels of E2 or E1 can be increased by cytochrome P450 (CYP)19A1-mediated formation form androgenic precursors, interconversion of E1 and E2 by hydroxysteroid 17-beta dehydrogenases (HSD17Bs), as well as sulfotransferase (STS)- and glucuronidase beta (GUSB)-mediated hydrolysis of estrogen sulfates and glucuronides, respectively. Tissue levels of E1 and E2 can be decreased by hydroxylation catalyzed by CYPs and conjugation, i.e., sulfonation and glucuronidation by sulfotransferases (SULTs) and UDP-glucuronosyltransferases (UGTs), respectively. However, only E1-G (Pemp et al. 2019a) and E1-S have been detected in breast GLT and ADT up to now. Catecholic hydroxy-estrogens (HO-E) can be oxidized to potentially cancer-initiating estrogen quinones which can be reduced back to catechols by NADPH quinone dehydrogenase 1 (NQO1). Detoxification of catechols is catalyzed by catechol-O-methyl transferase (COMT) resulting in the formation of methoxy(MeO-)estrogens. Of all possible MeO-estrogens, only 2-MeO-E1 has been detected mass spectrometrically (Fleming et al. 2010; Pemp et al. 2019a), predominately in ADT (Pemp et al. 2019a). Framed estrogens were quantified recently in breast tissues (Pemp et al. 2019a) and are used in this study as dependent variables in multiple linear regression analyses. Gray-colored estrogens were below the limit of detection in breast tissues (Pemp et al. 2019a)

Only recently, we described quantitative estrogen profiles and transcript levels of enzymes involved in E2 (biotrans)formation in breast GLT and ADT of pre- and postmenopausal women without breast cancer (Pemp et al. 2019a; Fig. 1) providing suitable data to determine variables affecting intramammary levels of estrogens. Furthermore, it was shown that levels of most estrogens and ratios thereof as well as levels of transcripts encoding enzymes involved in their (biotrans)formation differed significantly between GLT and ADT (Pemp et al. 2019a), demonstrating that breast GLT and ADT should be considered separately. Thus, the aim of the present study was to identify variables (reproductive history, lifestyle, and transcript levels of enzymes involved in intracrine activity) influencing levels of estrogens and ratios thereof in breast GLT and ADT.

## Materials and methods

### Origin of biospecimens

Breast tissue specimens were obtained from 47 adult women without breast cancer undergoing reduction mammoplasty between 2010 and 2015. All women participating in the study gave their written informed consent prior to their inclusion in the study. Women with a personal and/or family history of breast cancer were not eligible for participation. Information on age, height, weight, parity (parous/nulliparous), smoking habits (never smoker, current smoker, current nonsmoker, and the latter two with daily cigarette consumption) was volunteered by 47 women, and information on the intake of EADs by 45 women. Body mass index (BMI) was calculated in kg/m^2^.

### Sample preparation and characterization

Biospecimens were prepared as described previously (Pemp et al. 2019a). Briefly, aliquots of apparently plain ADT and GLT with less than 15% adhering ADT were flash-frozen in liquid nitrogen and stored at − 80 °C. From mixed tissues, GLT was isolated from cryosections (40 µm) at maximum − 20 °C using a scalpel. Biospecimens were characterized by their mass percentages of oil (oil%), percentage of area covered by intra- and interstromal adipocytes, and lobule type: oil% in GLT and ADT were determined gravimetrically after extraction with chloroform. Percentage of area covered by intra- and interstromal adipocytes was estimated microscopically (Leica LMD6500) in cryosections (10 µm) of GLT stained with hematoxylin and eosin Y by two different persons and coded slides (Pemp et al. 2019a). The lobule type of GLT was determined microscopically according to Russo and Russo (2004) and Figueroa et al. (2014).

### Instrumental analysis of E2, estrone, 2-methoxy-estrone, estrone sulfate, and glucuronide

E2, E1, and 2-methoxy(MeO)-E1 were determined by GC–MS/MS (Varian 450-GC, 300-MS; Bruker Daltonics, Bremen, Germany), whereas E1 sulfate (E1-S) and E1 glucuronide was determined by LC–MS/MS (QTrap^®^ 5500; AB Sciex, Darmstadt, Germany). Tissue levels of E2, E1, 2-MeO-E1, and E1-S were quantified using their respective deuterated derivatives (Pemp et al. 2019a). Data used in statistical analyses are presented in Online Resource 2.

### Determination of transcript levels

Analysis of transcript levels of genes encoding enzymes involved in E2 (biotrans)formation and regulation thereof was performed using customized Taqman^®^ Low Density Arrays and Taqman^®^ Gene Expression Assays as described by Pemp et al (2019a). Data used in statistical analyses are presented in Online Resource 3.

### Statistical methods

All statistical analyses were performed with the statistical programming language R [https://www.R-project.org/], version 3.5.2, and all tests of statistical significance were two-sided. Whenever multiple comparisons were performed, *p* values were adjusted using Holm’s method.

#### Contingency analyses and linear regression models

Contingency analyses were performed using Chi-square test. In case of categories following a natural order, Chi-square test for trend was used.

To test the association of every possible explanatory variable (exVAR) with the dependent variable, the variable explaining the dependent variable best is chosen by an automatic procedure. Subsequently, all possible exVARs are added one after another to the first one, ultimately choosing the one improving the model most, applying the Akaike information criterion. This is repeated until the model cannot be further improved by adding exVARs. Thus, each exVAR selected into the model contributes to modeling the dependent variable. The significance of the association is expressed by *p* values and the magnitude of impact is expressed by coefficients of regression. The choice of exVARs is discussed in the results section and more detailed information is given in Online Resource 4. If levels of estrogens or transcripts were below limit of quantification (LOQ) in > 40% of samples (Online Resources 2 and 3), they were not included as exVARs.

For levels of transcripts or estrogens below the respective limit of detection (LOD) and below the respective LOQ, LOD and LOQ were set, respectively. When levels of transcripts or estrogens were < LOQ in > 1 sample and ≤ 40% of samples, the levels of the respective transcript/estrogen were included additionally to the continuous exVAR as the qualitative presence of the exVAR (binary exVARq, compared to levels < LOQ). If, in the computed model, observations with Cook’s Distance > 1 appeared, they were removed and the model was computed anew. This process was repeated until no conspicuous observations occurred. To achieve normal distribution, dependent variables were logarithmized. Data distributions were evaluated in Quantile–Quantile plots with simulated confidence bands. Constant standard deviations of the errors were evaluated using scale-location plots. To check the model assumption of independent identically distributed errors, the residual vs. fitted values plot was used.

The adjusted coefficients of determination, the numbers of conspicuous observations removed, the numbers of observations contributing to the final models (maximum of 45 because of two specimens without information on the intake of EADs). In models considering intracrine activity, maximum number of observations was further reduced because of two and one specimens in GLT and ADT, respectively, without information on transcript levels), and the ratio of observations per exVAR of each final model is given. To achieve accurate estimation of regression coefficients, at least two observations per exVAR (Austin and Steyerberg 2015) were aimed for.

In addition, the regression coefficients (which represent the mean changes in the dependent variables for one unit of change in the respective exVAR while holding other predictors in the models constant), their confidence interval, as well as the *p* values of each exVAR selected are given in Online Resource 4.

#### Analyses of independence of variables

Spearman’s rank correlation analysis was performed to identify collinearity between numerical exVARs which might hinder each other selection and/or influence each other *p* values within the models. In the case of variables with > 1 level below LOD or LOQ, correlation was calculated with randomly distributed ranks for ties 10,000 times and highest Spearman correlation coefficients and lowest *p* values were used to rather overestimate collinearity. Relationship between categorical and numerical exVARs was evaluated by comparison of medians using unpaired Wilcoxon tests. Indications for relationships between variables and possible consequences for the selection of exVARs are given for each model in Online Resource 4.

## Results and discussion

To identify exVARs (e.g., breast cancer risk factors) influencing dependent variables (e.g., tissue levels of E2) by multiple linear regression models, suitable dependent variables were identified first and potential exVARs were chosen subsequently. Then, multiple linear regression models using stepwise forward selection were applied to assess up to 32 exVARs possibly influencing tissue levels of estrogens and ratios thereof in GLT and ADT.

### Identification of dependent variables

E2, E1, and E1-S were detected in most biospecimens, whereas 2-MeO-E1 was detected predominately in ADT (Online Resource 2). Thus, the influence of exVARs on levels of E2, E1, E1-S, and ratios thereof was analyzed in both GLT and ADT whereas the influence of exVARs on levels of 2-MeO-E1 could be analyzed in ADT only. Furthermore, continuous variables significantly influencing estrogen levels were further analyzed as dependent variables as well.

### Identification of possible exVARs related to the study cohort and to the tissues collected

First, the study cohort and the tissues collected were characterized and possible exVARs were defined.

#### Age, reproductive history, and related variables

**Fig. 2:**
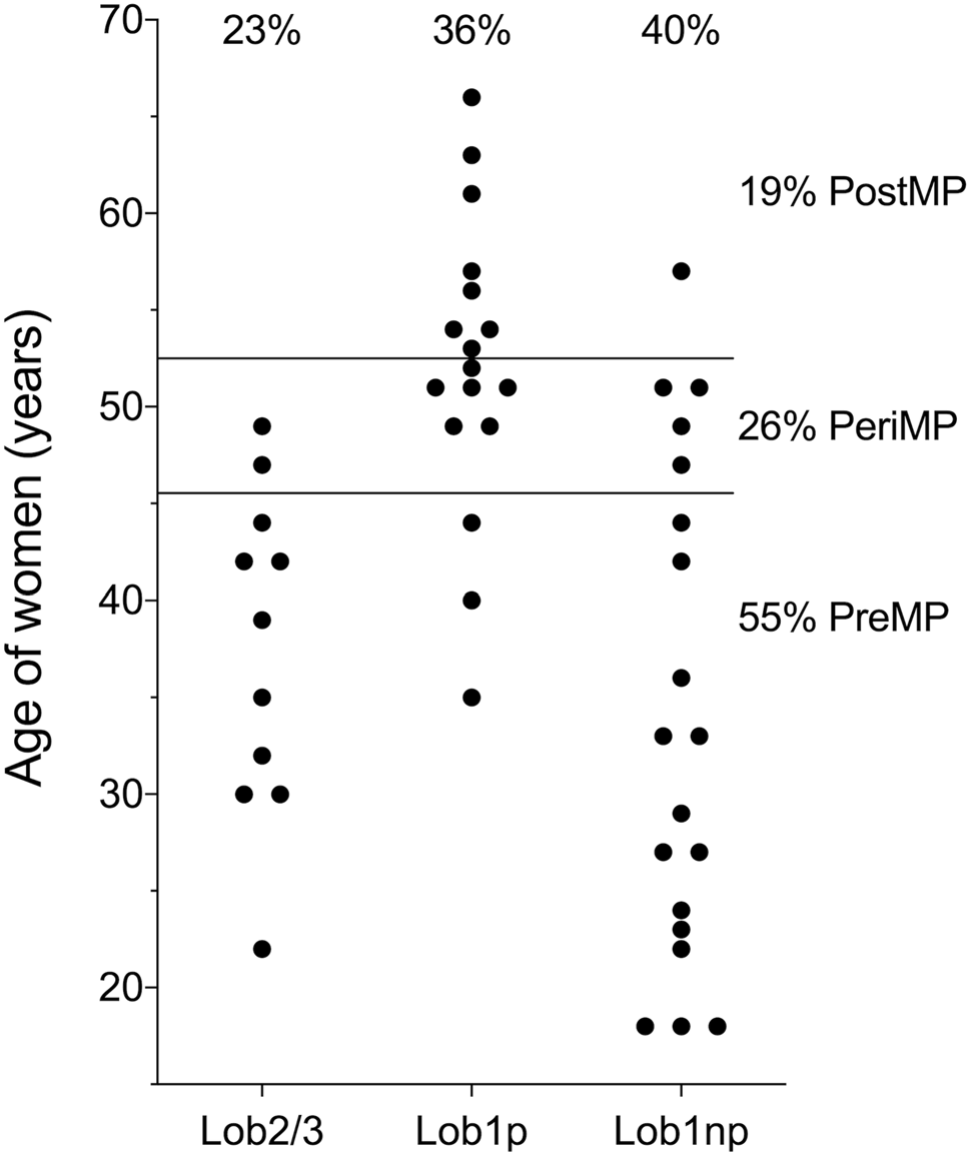
Distribution of age and lobule type as well as allocation of menopausal status (MP) of the women contributing specimens to the present study. GLT of parous women exhibits lobule type 2/3 (Lob2/3) until age-related regression. Lob1 predominates in parous women after age-related regression as well as in nulliparous (np) women (see text)

The age of the 47 women participating in the study ranged from 18–66 years. Most tissues were derived from women in the age group of 40–49 years (Online Resource 5). Menopausal status of the study population was allocated according to the range of age at menopause (46–52 years) observed in German women participating in the EPIC study (*n* > 27,000; Tsilidis et al. 2011) instead of assessment based on the women’s menstrual cycle characteristics (information not available). Thus, with a high probability, women > 52 and < 46 years old can be assumed to be post- (19%, Fig. 2) and premenopausal (55%), respectively. Women between 46–52 years were grouped as perimenopausal (26%). However, this group is likely to contain pre-, peri-, and postmenopausal women. To reflect continuous influence of age as well as abrupt influence of menopause on dependent variables, both the potential exVARs age and menopausal status were included into the models.

Regarding reproductive history, 40% of the women participating in the study were nulliparous. In the age class 35–54 years old, 27% were nulliparous, compared to 21% in the respective general population (Online Resource 5). The study population was further classified regarding parity and lobule type of the GLT, the latter reflecting age- and parity-related histological changes within the breast. These changes are most obvious in parous women, where lobules type 2 and 3 (Lob2/3), previously induced during pregnancy, regress back to lobule type 1 (Lob1p; Russo and Russo 2004). Nulliparous women (exhibiting lobule type 1, Lob1np) represented the third group of lobule types. 23%, 36%, and 40% of GLT were classified Lob2/3, Lob1p, and Lob1np, respectively (Fig. 2). In the following linear regression models, lobule type was tested as categorial exVAR. Relationship of lobule type with menopausal status and age cannot be completely excluded, but seems to be unlikely (Fig. 2).

#### Obesity and related variables

According to the WHO BMI classification, 53% and 13% of women were pre-obese and obese, respectively. The remaining women (34%) were of normal weight (Online Resource 6). Thus, compared to the German adult female population (29% pre-obese, 24% obese, and 45% of normal weight; Mensink et al. 2013), a higher percentage was pre-obese, but lower percentages were obese and of normal weight. BMI was included as continuous exVAR into the linear regression models.

**Fig. 3:**
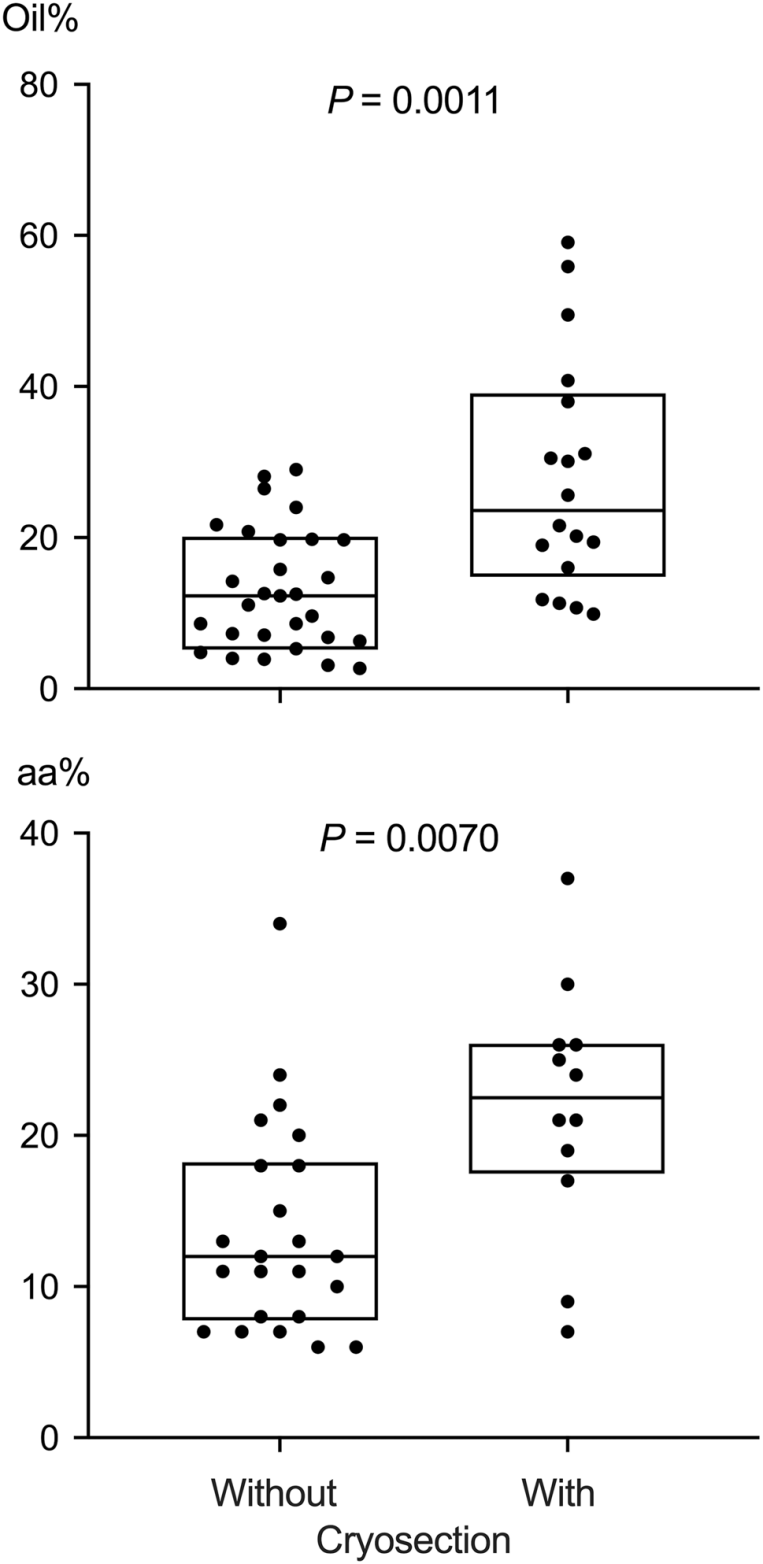
Comparison of oil% and area covered by adipocytes (aa%) in GLT isolated with and without cryosection. For statistical comparison of medians, unpaired Wilcoxon test was used. Boxplots depict 25th percentile, median, and 75th percentile. *P* values were adjusted for multiple comparisons (*n* = 2) using Holm’s method

Median oil% in GLT and ADT were 16% and 85%, respectively (Pemp et al. 2019a). In the following linear regression models, oil% were included as continuous exVAR. Directly isolated GLT and GLT isolated from mixed tissue were compared by means of two variables, oil% and relative areas covered by inter- and intrastromal adipocytes in GLT, and *p* values were adjusted for two comparisons. Despite detaching adhering ADT, GLT isolated from mixed tissues (*n* = 18) still exhibited significantly higher oil% (Fig. 3) than GLT which could be isolated directly; indicating a higher number and/or size of adipocytes within intra- and interlobular stroma. Consistently, microscopic comparison of GLT isolated directly and GLT isolated from mixed tissues revealed a significantly larger relative area covered by inter- and intrastromal adipocytes in GLT derived from mixed tissues (Fig. 3). Thus, in the following sections, these specimens will be referred to as large-adipocyte-area (laa)GLT and small-adipocyte-area GLT. The occurrence of laaGLT was statistically independent of BMI classification of the women donating the tissues (*p* = 0.40, Chi-square test for trend) and lobule types of GLT specimens (*p* = 0.28, Chi-square test, Online Resource 7). The occurrence of laaGLT was tested as binary exVAR.

#### Smoking and intake of EADs

Twelve women (26%) declared being current smokers (2–25 cigarettes/day) and EADs were used by eight women. These information were included as categorial exVARs into the linear regression models.

Mosaic plots characterizing the study population used in the linear regression models regarding the exVARs menopausal status, lobule type, BMI, intake of estrogen-active drugs, and smoking are depicted in Online Resource 8.

### Identification of possible exVARs related to estrogen biotransformation in tissues

Further possible continuous exVARs were tissue levels of the direct precursor estrogen(s) and of transcripts encoding enzymes directly forming or further metabolizing the dependent variable according to Fig. 1. In models with ratios of levels of different estrogens as dependent variables, further exVAR considered were levels of transcripts encoding enzymes directly forming or further metabolizing at least one of the estrogens involved in the ratio. If levels of transcripts or precursor estrogens were < LOQ in > 1 sample and ≤ 40% of samples, the qualitative presence of the respective precursor estrogen or transcript was included as binary exVARq, as well.

### Variables influencing tissue levels of estrogens identified by multiple linear regression models

Previous studies investigating variables associated with levels of estrogens in breast tissues either performed no statistical analysis at all or univariate analysis (i.e., comparisons of medians in case of categorical variables and correlation analyses in the case of continuous variables, Online Resource 1). In addition, methods nowadays less recommended for biospecimen analysis (Labrie et al. 2015) were applied to determine estrogen levels and/or undefined specimens were used without specifying the presence of GLT or ADT. Because of these differences, the outcome of the present study is only compared with previous ones if at least either a specific method or specifically GLT or ADT was used. Moreover, results observed in previous studies using tissues derived from women with breast cancer or from both women with and without breast cancer together for statistical analyses were included in Online Resource 1, but are not discussed in the following sections.

#### Age and menopause

**Fig. 4:**
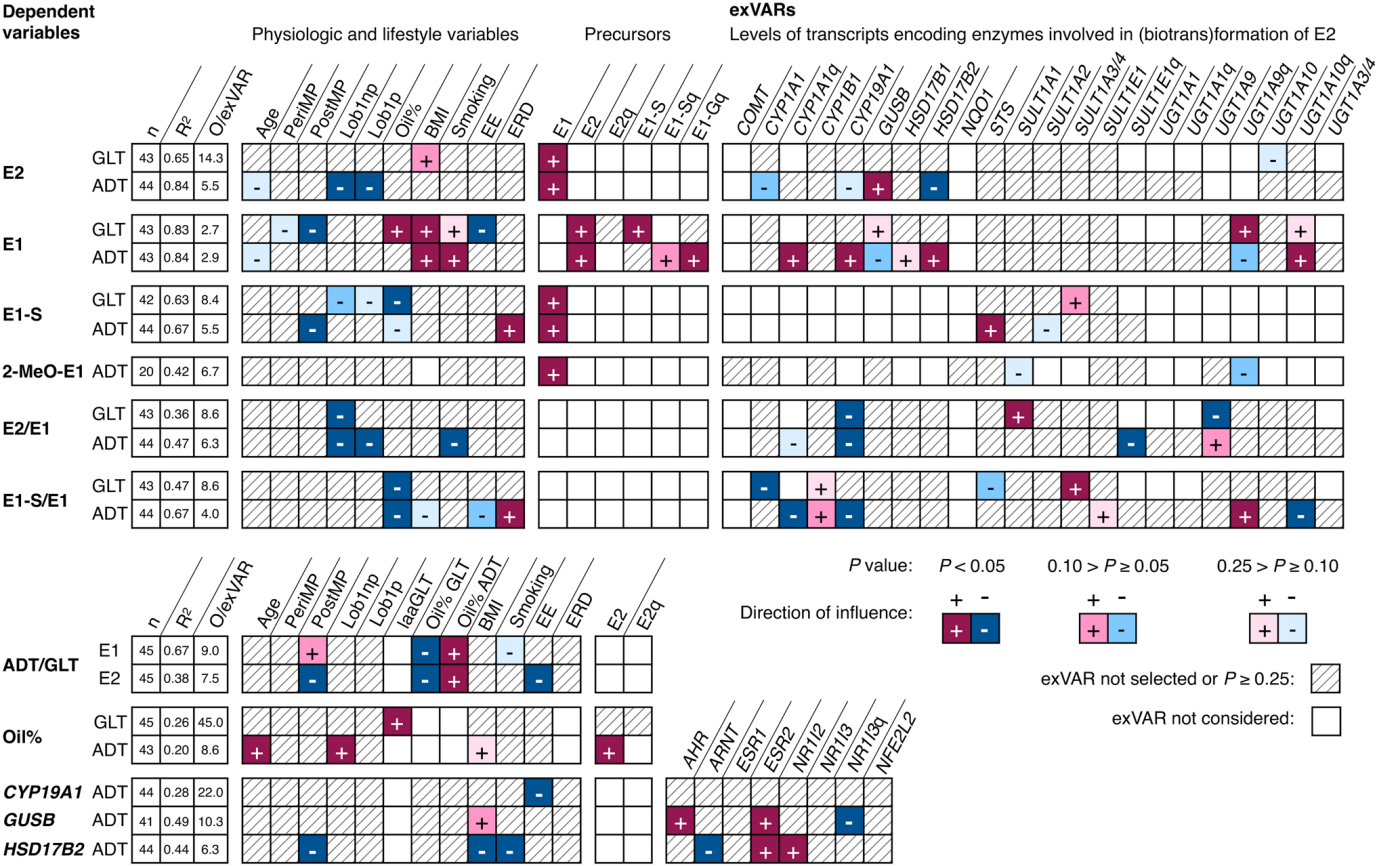
Influence of various exVARs on levels of estrogens as well as on ratios thereof and oil% in GLT and ADT (dependent variables) identified by multiple linear regression models using stepwise forward selection as detailed in Online Resource 4. For each model, the adjusted coefficient of determination (*R*^2^), and ratio of the number of observations (i.e., biospecimens) to exVAR (O/exVAR) after forward selection of variables is given

Cessation of ovarian estrogen production in menopause decreases blood levels of estrogens (Endogenous Hormones Breast Cancer Collaborative Group 2011, 2013) and is thus considered to affect levels of estrogens in breast tissues. Depypere et al. (2015) observed lower median levels of E2 in GLT derived from postmenopausal than from premenopausal women, yet no statistical analysis was performed. In the present study, levels of E2 were not directly influenced by postmenopausal status. However, postmenopausal status influenced levels of E1 in GLT and levels of E1-S in ADT negatively (*p* < 0.05). Furthermore, the ratio of E2 levels in ADT to E2 levels in GLT (ADT/GLT of E2) was also influenced negatively by postmenopausal status (*p* < 0.05). Interestingly, ADT/GLT of E1 was rather positively influenced by postmenopausal status (0.10 > *p* ≥ 0.05, Fig. 4). Thus, menopause seems not to affect levels of all estrogens in GLT and ADT and ratios ADT/GLT of estrogens in the same way which cannot be explained by a mere decrease in systemic delivery of estrogens via plasma. The continuous exVAR age did not directly influence levels of any estrogen or ratio thereof significantly (Fig. 4).

#### Lobule type

The impact of lobule type on estrogen levels has not been investigated yet. Average levels of E1-S in GLT exhibiting the least developed lobule type 1 (derived from nulliparous women, Lob1np, or from parous women after age-related regression, Lob1p) were lower than levels of E1-S in GLT categorized Lob2/3, yet not significantly (0.10 > *p* ≥ 0.05 and *p* > 0.1, respectively; Fig. 4). Furthermore, lobule type 1 influenced levels of E2 and E2/E1 in ADT negatively compared to Lob2/3 (*p* < 0.05, Fig. 4).

#### Oil%

Median oil% in GLT and ADT were 16% and 85%, respectively (Pemp et al. 2019a). Estrogen levels might be affected by oil% via: (i) physicochemical distribution of the lipophilic molecules E2 and E1 and the more hydrophilic E1-S and (ii) cell-specific enzyme expression in stromal adipocytes. Oil% influenced levels of E1 in GLT (positively, *p* < 0.05), levels of E1-S in GLT (negatively, *p* < 0.05), and E1-S/E1 in both GLT and ADT (negatively, *p* < 0.05; Fig. 4). Interestingly, levels of 2-MeO-E1 and E2 (which are more and comparably lipophilic than E1) were not influenced by oil% in ADT (Fig. 4), thereby rendering a mere physicochemical effect less likely. In line with the influence of oil% on the respective estrogens, ADT/GLT of both E2 and E1 were positively influenced by oil% in ADT but negatively in GLT (*p* < 0.05).

Since oil% influenced levels of some estrogens and ratios thereof in ADT and GLT, variables affecting oil% were investigated as well. Besides age, lifestyle factors, and tissue characteristics, oil% may be influenced by estrogens affecting lipogenesis and adipogenesis (Gao and Dahlman-Wright 2013), and thus, levels of E2 were also considered as exVAR. Furthermore, the classification of GLT regarding adipocyte area laaGLT was considered as exVAR as well. Oil% in GLT were positively influenced by laaGLT (*p* < 0.05), but no other exVAR was selected (Fig. 4). Interestingly, oil% in ADT were positively influenced by levels of E2, age, and specimens derived from nulliparous women (compared to parous women prior to age-related regression, all *p* < 0.05), and not significantly, by BMI (*p* > 0.1, with no apparent statistical reason such as collinearity interfering with the exVAR BMI, Online Resource 4).

Notably, both linear regression models exhibited low *R*^2^ values (Fig. 4) suggesting either important variables missing in the model (which seems likely in this case) or large variations within the data set.

#### Lifestyle-associated variables

A positive association between BMI and estrogens in serum has been observed in pre- (E2, E1; Endogenous Hormones Breast Cancer Collaborative Group 2013) and postmenopausal women (E2, E1, E1-S; Endogenous Hormones Breast Cancer Collaborative Group 2003, 2011). The common interpretation is that an increase in BMI leads to an increase in the mass of adipose tissue within the whole body, accompanied by a change in intra- and extramammary function of adipose tissue (Yaghjyan and Colditz 2011; Brown 2014). Consequently, a higher amount of estrogens is produced and distributed within the body via blood (Lønning et al. 2011), contributing to estrogen levels in breast tissue. Yet, no studies investigating associations between BMI and estrogen levels in breast tissues derived from women without breast cancer have been identified. In the present study, BMI influenced levels of E2 in GLT (0.10 > *p* ≥ 0.05) as well as E1 in GLT and ADT (*p* < 0.05) positively. In contrast, tissue levels of E1-S were not influenced by BMI (Fig. 4).

Besides BMI, smoking has also been associated with higher levels of E2 and E1 in blood of postmenopausal women (Endogenous Hormones Breast Cancer Collaborative Group 2011) but not of premenopausal women (Endogenous Hormones Breast Cancer Collaborative Group 2013). No studies investigating the impact of smoking on levels of estrogens in breast tissues have been identified. In the present study, smoking influenced levels of E1 in ADT positively and, congruently, E2/E1 in ADT negatively (both *p* < 0.05, Fig. 4).

In all studies analyzing estrogens in breast tissues of women without breast cancer, the intake of EADs for oral contraception or hormone replacement therapy was either an exclusion criterium or not considered in statistical analyses (Online Resource 1). In the present study, the intake of ethinyl estradiol did not influence the levels of E2 in GLT or ADT (Fig. 4). Yet, intake of ethinyl estradiol influenced ADT/GLT of E2, levels of E1 in GLT (*p* < 0.05), and E1-S/E1 in ADT negatively (0.10 > *p* ≥ 0.05). In contrast, intake of E2-releasing drugs (containing E2 or E2 valerate) used for hormone replacement therapy positively influenced levels of E1-S and E1-S/E1 in ADT (*p* < 0.05), but did not influence levels of E2 or E1 in GLT or ADT.

#### Biotransformation precursors

No studies investigating correlations among estrogens in breast tissues derived from women without breast cancer have been identified. In the present study, biotransformation precursors of the respective estrogens influenced tissue levels of E2, E1, E1-S, and 2-MeO-E1 positively (*p* < 0.05, except for E1-S as precursor for E1 in ADT 0.10 > *p* ≥ 0.05; Fig. 4). Thus, exVARs affecting levels of, e.g., E1, may indirectly contribute to levels of E2, E1-S, and 2-MeO-E1. Interestingly, whereas levels of E1 in GLT were positively influenced by levels of E1-S, levels of E1 in ADT were positively influenced by the qualitative presence of both E1-G (*p* < 0.05) and E1-S (0.10 > *p* ≥ 0.05).

#### Transcripts encoding enzymes involved in estrogen (biotrans)formation

Savolainen-Peltonen et al. (2014) observed no correlation of levels of *CYP19A1,* i.e., aromatase, *STS,* and *HSD17B1,* with levels of E2 in ADT and the respective transcript levels did also not significantly influence levels of E2 in ADT in the present study. However, in the present study, levels of E2 in ADT were influenced positively by levels of the transcript encoding GUSB (Fig. 4, *p* < 0.05), the enzyme hydrolyzing estrogen glucuronides (Fig. 1). Estrogen glucuronides are mostly associated with elimination from tissues and body but may also contribute to intratissue levels of estrogens, even though GUSB and substrates only meet in a highly controlled manner (Naz et al. 2013). Furthermore, levels of E2 in ADT were influenced negatively (*p* < 0.05) by levels of transcripts encoding HSD17B2, the enzyme forming E1 by oxidation of E2 (Fig. 1). Congruently, levels of E1 in ADT were positively influenced by levels of *HSD17B2*. Moreover, levels of *CYP19A1* influenced levels of E1 in ADT positively (Fig. 4, *p* < 0.05). The presence of transcripts encoding the conjugating enzyme UGT1A9 and levels of transcripts encoding CYP1A1 influenced the levels of the respective substrates (i.e., E1, 2-MeO-E1, and E2, respectively) in ADT negatively (0.10 > *p* ≥ 0.05). Likewise, levels of *SULT1A3/4* influenced E1-S in GLT positively (0.10 > *p* ≥ 0.05). Forward selection of exVARs into models describing estrogen levels in ADT and GLT identified levels of further transcripts, yet the associations exhibited *p* values ≥ 0.10 (Fig. 4).

Regulation of transcription of genes encoding enzymes by the respective substrates (up-) and products (down-), is a common biochemical feedback mechanism. Furthermore, ligand-activated transcription factors are involved in the regulation of transcription of genes of biotransforming enzyme families; e.g., CYP (Tralau and Luch 2013), SULT (Runge-Morris et al. 2013), and UGT (Hu et al. 2014). Positive associations of levels of *STS* as well as presence of *UGT1A9* and *UGT1A10* were observed with the respective substrates of the encoded enzymes; i.e., E1-S in ADT, E1 in GLT and E1 in ADT, respectively (*p* < 0.05; Fig. 4). *UGT1A9* (Cho et al. 2016) and *UGT1A10* (Starlard-Davenport et al. 2008) may be regulated by activated ESR1, whereas STS may be regulated via G-protein-coupled ESR action (Gilligan et al. 2017).

Since levels of *CYP19A1, GUSB*, and *HSD17B2* influenced levels of estrogens in ADT significantly, exVARs influencing these variables were investigated, as well. Besides exVARs associated with physiology and lifestyle, levels of transcripts known to be directly or indirectly involved in regulation of phase I and phase II biotransformation were included, since little is known about specific regulation of the transcription of the genes encoding these enzymes (Naz et al. 2013; Zhao et al. 2016; Hilborn et al. 2017).

Levels of *CYP19A1* in ADT were exclusively influenced by the intake of ethinyl estradiol (negatively, Fig. 4, *p* < 0.05). It is known that the ovarial synthesis of estrogens is negatively regulated by estrogen-active compounds (Fleischman et al. 2010). Furthermore, CYP19A1 was detected less frequently in the endometrium of women taking oral contraceptives containing ethinyl estradiol than in non-users (Maia et al. 2013). Low *R*^2^ value of the model (Fig. 4) suggests at least one other important variable missing in the model (e.g., transcript levels of glucocorticoid receptor; Zhao et al. 2016).

Levels of *GUSB* in ADT were significantly influenced by levels of *AHR*, *ESR2* (positively), and qualitative presence of *NR1I3* (negatively). Binding sites for transcription factors such as Sp1 and AP-2 (Naz et al. 2013) in the promotor of the GUSB gene provide a link to estrogen signaling (Pellikainen and Kosma 2007; Safe and Kim 2008). BMI influenced levels of *GUSB* positively (0.10 > *p* ≥ 0.05). Most interestingly, levels of *HSD17B2* in ADT were significantly influenced by BMI, smoking, and postmenopausal status as well as by levels of *ARNT* (negatively) and levels of *ESR2* and *N1I2* (positively). Transcription of the *HSD17B2* gene is regulated by retinoic acid via RAR alpha/RXR alpha tethered to transcription factors Sp1 and Sp3 on the *HSD17B2* promoter (Cheng et al. 2008), which provides a link to ESR-mediated signaling. An obvious link between ARNT or N1I2 and expression of *HSD17B2* has not been described yet.

Concluding this section, it should be emphasized that lack of influence of transcript levels does not exclude the contribution of the respective enzyme activities to estrogen levels.

### Relevance

Including E2, E1, E1-S, and 2-MeO-E1, the present study encompasses the major estrogens detectable in breast tissues derived from women without breast cancer. In contrast to all previous studies, the present study fulfilled published prerequisites (Yaghjyan and Colditz 2011; Sherman et al. 2012; Rosner et al. 2013) for data acquisition concerning sample characterization as well as specificity and reliability of estrogen analysis (Pemp et al. 2019a).

However, mammoplasty specimens raise concern regarding sample bias, in particular (i) “young age”, (ii) “obesity”, and (iii) “extremely large fatty breasts” (Sherman et al. 2012), thus putatively reflecting a specific subpopulation. Most specimens were derived from women 40–49, which is also the modal age group of the respective general population (Online Resource 5). Yet, the study population indeed lacked women older than 66 years and the proportion of pre-obese and obese women was higher and lower than in the general population, respectively (Online Resource 6). Moreover, 38% of specimens were classified as laaGLT and exhibited higher oil% than GLT which could be isolated directly (section “Obesity and related variables”). Since no data on the occurrence of laaGLT in the general female population are available, it is currently unknown whether women undergoing mammoplasty are predisposed to laaGLT. Oil% significantly influenced levels of E1, E1-S in GLT, E1-S/E1 in GLT and ADT, as well as ADT/GLT of E1 and E2 and should, therefore, be considered in sample characterization of human breast biospecimens. Of note, oil% in GLT were not influenced by any exVAR deducible by questionnaire.

Intake of exogenous estrogens, menopausal status, BMI, and smoking were previously shown to be associated with levels of estrogens in blood (Endogenous Hormones Breast Cancer Collaborative Group 2003,2011; Fleischman et al. 2010, 2013; Gaudet et al. 2017). The general consensus is that blood levels of estrogens contribute to breast tissue levels, yet whether levels of circulating estrogens serve as surrogate for levels of estrogens in breast tissues, or more precisely, in GLT or ADT, is a matter of debate (Lønning et al. 2011; Bulun et al. 2012; Colditz and Bohlke 2014; Labrie 2015; Stanczyk et al. 2015; Vihma et al. 2016).

**Fig. 5:**
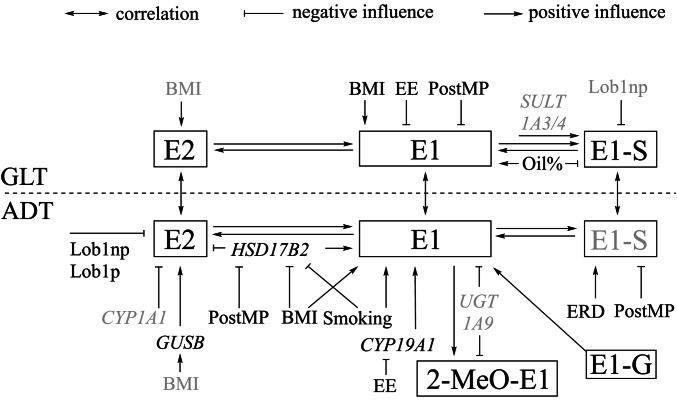
ExVARs associated with intramammary biotransformation pathways, lifestyle, and reproductive history influencing levels of estrogens (framed) as well as transcript levels of CYP19A1, GUSB, HSD17B2 in ADT with *p* < 0.05 (exVARs written in black color) or 0.10 > *p* ≥ 0.05 (exVARs written in grey color) identified by multiple regression models using stepwise forward selection. Correlations between estrogen levels in GLT and ADT determined by Spearman correlation analyses were described in Pemp et al. (2019a). *EE* ethinyl estradiol, *ERD* E2-releasing drug, *PostMP* postmenopausal status

In the present study, variables known to affect levels of circulating estrogens indeed influenced estrogen levels in breast tissues, as well (Fig. 5).

However, these exVARs affecting levels of circulating estrogens by targeting ovarial and adiposal production of estrogens did obviously not suffice to explain the variances in estrogen levels within the breast. In addition, estrogen levels in both GLT and ADT were further influenced by levels or the presence of their precursor estrogens and levels of transcripts encoding enzymes involved in estrogen biotransformation. Interestingly, whereas (as expected, Mueller et al. 2015) E1-S seems to represent a source of E1 in breast GLT, intratissue levels of E1 in ADT seem additional to be regulated by glucuronidation via UGT1A9 (Fig. 5). Furthermore, although E2-3-G was not detected in human breast tissues, in the light of the contribution of levels of *GUSB* to levels of E2 in ADT and a ten times higher LOD for E2-3-G compared to E1-G (Pemp et al. 2019a), E2 glucuronides cannot be excluded to represent a source of estrogens for the human breast, as well.

Further supporting the role of intratissue biotransformation, levels of E2 and of E1 in ADT were also significantly influenced by levels of *HSD17B2*. Recently, it was shown that levels of *Hsd17b* enzymes significantly influenced intramammary levels of E2 in the ACI rat model of tumorigenesis (Pemp et al 2019b), as well, supporting the important role of HSD17Bs in intramammary estrogen homeostasis (Hilborn et al. 2017) across species.

Furthermore, the present study not only supports the commonly accepted importance of CYP19A1 in estrogen homeostasis, but suggests its role within the breast tissue in addition to a systemic effect. Avoiding indirect associations in linear regression models, the influence of exVARs present in ADT on levels of E2 in GLT was not tested in the present study. However, due to correlations between estrogen levels in GLT and ADT (Pemp et al. 2019a) and the influence of precursor estrogens on the respective estrogen levels shown in the present study, levels of E2 in GLT might be affected indirectly by any variable significantly influencing E1 in ADT, i.e., levels of *CYP19A1, HSD17B2,* presence of *UGT1A9,* E1-S, and E1-G, as well as BMI and smoking (Fig. 5).

Inhibition of CYP19A1 suggested in breast cancer prevention (Advani and Moreno-Aspitia 2014; Colditz and Bohlke 2014) could thus lower intratissue E2 levels not only by systemic but also by intracrine mechanisms. However, systemic alteration of E2 biosynthesis harbors the risk of losing its beneficial biological effects, e.g., in bone health (Advani and Moreno-Aspitia 2014). Thus, drugs in development for the treatment of endocrine disorders by targeting enzymes involved in more organ-specific E2 homeostasis (e.g., inhibitors of HSD17B2 and STS; Konings et al. 2018) may also be successful in breast cancer chemoprevention.

In conclusion, a thorough characterization of specimens enabled taking into account variables related to obesity and “extremely fatty breasts” during statistical analyses. Tissue characterization of GLT derived from mammoplasty (and possibly also of biopsy) specimens by oil% as well as by lobule type seems to be advisable to prevent sample bias.

Novel insights in estrogen homeostasis in the normal human breast GLT and ADT support contribution of variables affecting both extra- and intratissue (biotrans)formation of estrogens and suggest a central role of E1 levels in breast ADT homeostasis. The susceptibility of estrogen homeostasis to systemic and tissue-specific modulation renders both beneficial and adverse effects of further variables associated with lifestyle and the environment possible.

## Electronic supplementary material

Below is the link to the electronic supplementary material.

Supplementary file1 (PDF 181 kb)

Supplementary file2 (PDF 434 kb)

Supplementary file3 (PDF 416 kb)

Supplementary file4 (PDF 828 kb)

Supplementary file5 (PDF 2830 kb)

Supplementary file6 (PDF 1924 kb)

Supplementary file7 (PDF 3152 kb)

Supplementary file8 (PDF 401 kb)

## Data Availability

The datasets generated during and/or analyzed during this study are included in this published article [and its supplementary information files] or are available from the corresponding author on reasonable request.
