# Supplementary material for: Influence of breast cancer risk factors and intramammary biotransformation on estrogen homeostasis in the human breast
Source: Arch Toxicol. 2020 Jun 22;94(9):3013–25. doi: 10.1007/s00204-020-02807-1 (PMC7415756; doi:10.1007/s00204-020-02807-1)
Supplement: Supplementary file 1 — Supplementary file1 (PDF 181 kb) [file 204_2020_2807_MOESM1_ESM.pdf]

Archives of Toxicology

Influence of breast cancer risk factors and intramammary biotransformation on estrogen homeostasis in the human breast

Daniela Pemp, Leo N. Geppert, Claudia Wigmann, Carolin Kleider, René Hauptstein, Katja Schmalbach, Katja Ickstadt, Harald L. Esch, Leane Lehmann*

***Corresponding author:**
Prof. Dr. Leane Lehmann, Chair of Food Chemistry, University of Würzburg, Am Hubland, D-97074 Würzburg, Germany. Phone: +49 931 318-5481. Email: leane.lehmann@uni-wuerzburg.de.

Online Resource 1 Literature data on the impact of breast cancer (BC) risk factors and mammary transcript levels of enzymes involved in (biotrans)formation of estrogens on breast tissue levels of estrogens; i.e. 17β-estradiol (E2), estrone (E1) and E1 sulfate (-S), in human breast glandular (GLTs) and adipose (ADTs) tissues of women without (w/o) or with (w) BC.

Statistical analyses consisted of (i) comparing differences in distribution between groups by Kruskal-Wallis followed by comparison of medians by Dunn's multiple comparison (D) and (ii) correlation analyses by Pearson product-moment correlation (PC) or Spearman's rank correlation analysis (SC)

+, positive correlation;

(+), positive correlation but statistically not significant; i.e., 0.1 > *P* > 0.05)

0, no correlation;

?, not reported or not analyzed;

BT, breast tissue;

mix, data of women without and with breast cancer were combined and then statistically analyzed;

MP, menopausal status;

n, number of individuals participating in the study;

n.s., not specified

pre, premenopausal; post, postmenopausal;

BT	BC	MP	n	Estrogen	BC risk factor	Intracrine activity	Statistical analysis	Reference
GLT	w/o	pre	37[1]	E2	EAD: w (n=12)[A] < w/o (n=25)		none	Depypere et al. 2015
ADT	w/o	post	14[1A]	E2	age		0 SC	Savolainen-Peltonen et al. 2014
				E2	age at menarche and menopause, BMI, number of labours, history of EAD use		?	
				E2		HSD17B12	(+) SC	
				E2		CYP19A1, STS, HSD17B1, 7	?	
ADT	mix	pre	28[1A]	E2, E1	age, age at menarche, BMI, number of labours, history of EAD use		?	Savolainen-Peltonen et al. 2018
				E2		CYP19A1, STS, HSD17B12	0 SC	
						HSD17B1, 7	?	
				E1		CYP19A1, STS, HSD17B12	+ SC	
						HSD17B1, 7	?	
ADT	mix	post	24[2]	E1	BMI		+ SC	Vihma et al. 2016
ADT	w	post	69[3]	E2	BMI		0 PC	Falk et al. 2012
			31[3]	E2	BMI		0 PC	
			69[3]	E1	BMI		+ PC	
			31[3]	E2	BMI		+ PC	
			69[3], 31[3]	E2, E1	age, age at menarche and menopause, BMI, number of pregnancies, age at first birth		?	
ADT	w	post	35[3]	E2	BMI		0 PC	Szymczak et al. 1998
				E1	BMI		?	
				E1S	BMI		+ PC	
ADT	w	post	14[1A]	E2	age		+ SC	Savolainen-Peltonen et al. 2014
					age at menarche and menopause, BMI, number of labours, history of EAD use		?	
						HSD17B12	0 SC	
						CYP19A1, STS, HSD17B1, 7	?	
ADT	?	pre	36[1]	E2	EAD: w (n=7)[B] < w/o (n=29)		D	O’Brien et al. 1997
		post	29[1]	E1	EAD: w (n=10)[C] > w/o (n=19)[4]		D	
		pre	36[1B]	E2, E1	BMI		0 PC	
n.s.	w	pre	13[4A]	E2, E1, E1S	BMI		0 SC	Lønning et al. 2009
		post	34[4A]	E2, E1S	BMI		0 SC	
		post	34[4A]	E1	BMI		+ SC	
		mix	34[4A]	E2		CYP19A1, STS, HSD17B1, 2, 5, 7, 12, 14	0 SC	Lønning et al. 2009; Haynes et al. 2010
		post	23[4A]	E2		CYP19A1	(+) SC	
				E2		STS, HSD17B1, 2, 5, 7, 12, 14	0 SC	

Notes:

[1], intake of EADs included

[2], intake or exclusion of EADs was not specified

[3], exclusion of women taking EADs

[A], chemical nature of the EAD was not specified

[B], ethinyl-E2 (all 7)

[C], conjugated equine estrogens (7), oral E2 (2), injectable E2 valerate (1)

[4], women did not take EADs or stopped 7-30 days (n=6, five women had had bilateral oophorectomies O’Brien et al. 1997), 4 weeks (Lønning et al. 2009), or 6 months (Haynes et al. 2010) prior to surgery

References

Depypere HT, Bolca S, Bracke M, Delanghe J, Comhaire F, Blondeel P (2015) The Serum Estradiol Concentration Is the Main Determinant of the Estradiol Concentration in Normal Breast Tissue. Maturitas 81:42-45. https://doi.org/10.1016/j.maturitas.2015.01.014

Falk RT, Gentzschein E, Stanczyk FZ, Garcia-Closas M, Figueroa JD, Ioffe OB, Lissowska J, Brinton LA, Sherman ME (2012) Sex Steroid Hormone Levels in Breast Adipose Tissue and Serum in Postmenopausal Women. Breast Cancer Res Treat 131:287-294. https://doi.org/10.1007/s10549-011-1734-5

Haynes BP, Straume AH, Geisler J, A'Hern R, Helle H, Smith IE, Lønning PE, Dowsett M (2010) Intratumoral estrogen disposition in breast cancer. Clin Cancer Res 16:1790-801. https://doi.org/10.1158/1078-0432.CCR-09-2481

Lønning PE, Helle H, Duong NK, Ekse D, Aas T, Geisler J (2009) Tissue Estradiol Is Selectively Elevated in Receptor Positive Breast Cancers While Tumour Estrone Is Reduced Independent of Receptor Status. J Steroid Biochem Mol Biol 117:31-41. https://doi.org/10.1016/j.jsbmb.2009.06.005

O'Brien SN, Anandjiwala J, Price TM (1997) Differences in the Estrogen Content of Breast Adipose Tissue in Women by Menopausal Status and Hormone Use. Obstet Gynecol 90:244-248. https://doi.org/10.1016/S0029-7844(97)00212-3

Savolainen-Peltonen H, Vihma V, Wang F, Turpeinen U, Hamalainen E, Haanpaa M, Leidenius M, Tikkanen MJ, Mikkola TS (2018) Estrogen biosynthesis in breast adipose tissue during menstrual cycle in women with and without breast cancer. Gynecol Endocrinol 34:1039-1043. https://doi.org/10.1080/09513590.2018.1474868

Savolainen-Peltonen H, Vihma V, Leidenius M, Wang F, Turpeinen U, Hamalainen E, Tikkanen MJ, Mikkola TS (2014) Breast Adipose Tissue Estrogen Metabolism in Postmenopausal Women with or without Breast Cancer. J Clin Endocrinol Metab 99:E2661-2667. https://doi.org/10.1210/jc.2014-2550

Szymczak J, Milewicz A, Thijssen JH, Blankenstein MA, Daroszewski J (1998) Concentration of Sex Steroids in Adipose Tissue after Menopause. Steroids 63:319-321.

Vihma V, Wang F, Savolainen-Peltonen H, Turpeinen U, Hamalainen E, Leidenius M, Mikkola TS, Tikkanen MJ (2016) Quantitative Determination of Estrone by Liquid Chromatography-Tandem Mass Spectrometry in Subcutaneous Adipose Tissue from the Breast in Postmenopausal Women. J Steroid Biochem Mol Biol 155:120-125. https://doi.org/10.1016/j.jsbmb.2015.10.004
