# Supplementary material for: Influence of breast cancer risk factors and intramammary biotransformation on estrogen homeostasis in the human breast
Source: Arch Toxicol. 2020 Jun 22;94(9):3013–25. doi: 10.1007/s00204-020-02807-1 (PMC7415756; doi:10.1007/s00204-020-02807-1)
Supplement: Supplementary file 2 — Supplementary file2 (PDF 434 kb) [file 204_2020_2807_MOESM2_ESM.pdf]

Archives of Toxicology

Influence of breast cancer risk factors and intramammary biotransformation on estrogen homeostasis in the human breast

Daniela Pemp, Leo N. Geppert, Claudia Wigmann, Carolin Kleider, René Hauptstein, Katja Schmalbach, Katja Ickstadt, Harald L. Esch, Leane Lehmann*

***Corresponding author:**
Prof. Dr. Leane Lehmann, Chair of Food Chemistry, University of Würzburg, Am Hubland, D-97074 Würzburg, Germany. Phone: +49 931 318-5481. Email: leane.lehmann@uni-wuerzburg.de.

Online Resource 2 Individual percentages of oil (oil%), levels of estrogens in breast glandular tissues (GLT, part A) and breast adipose tissues (ADT, part B) and ratios thereof (part C).

Biospecimens were listed using arbitrary numbers (No.). Limits of detection/quantification (LOD/LOQ) were: 17/40 (17β-estradiol, E2), 20/45 (estrone, E1), 35/100 (2-methoxy-E1, 2-MeO-E1), 19/35 (E1 sulfate, E1-S), and 250/n.d. (E1 glucuronide, E1-G) fmol/g. Furthermore, levels of the following analytes were below LOD in every sample: 2-HO-E2 (<178 fmol/g), 4-HO-E2 (<268 fmol/g), 2-hydroxy (HO)-E1 (<423 fmol/g), 4-HO-E1 (<470 fmol/g), 16α-HO-E2 (<1,693 fmol/g), 16α-HO-E1 (<39 fmol/g), 2-MeO-E2 (<26 fmol/g), 4-MeO-E2 (<21 fmol/g), 4-MeO-E1 (<62 fmol/g), E2-3-S (< ≈400 fmol/g), E2-17-S (< ≈5,000 fmol/g), E2-3-G (< ≈3,500 fmol/g).

<, below LOD or below LOQ or ratio calculated with LOD or LOQ of E2, 2-MeO-E1, and E1-S;

>, qualitative determination >LOD (E1-G)

Part A Individual percentages of oil (oil%) and levels of estrogens in breast GLT

GLT									
		Levels (fmol/g)					Ratios		
**No.**	**Oil%**	E2	E1	2-MeO-E1	E1-S	E1-G	E2/E1	2-MeO-E1/E1	E1-S/E1
1	20	236	357	<35	321	>250	0.66	<0.10	0.90
2	20	92	922	<100	1,788	>250	0.10	<0.11	1.94
3	24	193	541	<35	494	<250	0.36	<0.06	0.91
4	9	281	5,267	627	25,674	>250	0.05	0.12	4.87
5	5	44	95	<35	510	<250	0.46	<0.37	5.37
6	21	48	381	<35	321	<250	0.13	<0.09	0.84
7	38	138	235	<35	169	<250	0.59	<0.15	0.72
8	29	82	427	<35	528	<250	0.19	<0.08	1.24
9	15	109	217	<35	424	<250	0.50	<0.16	1.95
10	13	520	699	<35	4,503	>250	0.74	<0.05	6.44
11	10	947	1,953	298	1,016	>250	0.48	0.15	0.52
12	20	717	772	<35	1,597	>250	0.93	<0.05	2.07
13	11	<40	113	<35	40	<250	<0.35	<0.31	0.35
14	7	47	77	<35	291	>250	0.61	<0.45	3.78
15	28	1,035	1,172	376	1,659	>250	0.88	0.32	1.42
16	10	<17	269	<35	334	<250	<0.06	<0.13	1.24
17	4	467	943	<35	3,407	>250	0.50	<0.04	3.61
18	3	48	95	<35	274	<250	0.51	<0.37	2.88
19	16	50	231	<35	280	<250	0.22	<0.15	1.21
20	12	130	290	<35	525	<250	0.45	<0.12	1.81
21	50	61	435	<35	147	>250	0.14	<0.08	0.34
22	22	707	784	<35	928	>250	0.90	<0.04	1.18
23	11	190	536	<35	1,131	>250	0.35	<0.07	2.11
24	4	58	57	<35	325	>250	1.02	<0.61	5.70
25	12	205	405	<35	4,021	>250	0.51	<0.09	9.93
26	27	1,141	912	116	2,100	>250	1.25	0.13	2.30
27	14	<17	98	<35	208	<250	<0.17	<0.36	2.12
28	59	113	391	<35	247	<250	0.29	<0.09	0.63
29	56	283	1,018	173	244	<250	0.28	0.17	0.24
30	7	111	242	<35	280	<250	0.46	<0.14	1.16
31	19	353	1,009	<35	1,044	>250	0.35	<0.03	1.03
32	30	864	1,179	126	1,374	>250	0.73	0.11	1.17
33	13	122	249	<35	990	<250	0.49	<0.14	3.98
34	26	197	515	<35	164	<250	0.38	<0.07	0.32
35	5	139	182	<35	604	<250	0.76	<0.19	3.32
36	16	96	252	<35	209	>250	0.38	<0.14	0.83
37	41	98	525	<35	189	<250	0.19	<0.07	0.36
38	22	<40	238	<35	88	<250	<0.17	<0.15	0.37
39	3	69	154	<35	229	>250	0.45	<0.23	1.49
40	31	127	166	<35	62	<250	0.77	<0.21	0.37
41	19	355	708	<35	1,417	>250	0.50	<0.05	2.00
42	9	591	799	<35	1,412	>250	0.74	<0.04	1.77
43	7	71	95	<35	148	<250	0.75	<0.37	1.56
44	6	195	201	<35	207	<250	0.97	<0.17	1.03
45	31	496	632	<35	546	<250	0.78	<0.06	0.86
46	20	69	98	<35	310	<250	0.70	<0.36	3.16
47	11	49	62	<35	236	<250	0.79	<0.56	3.81

Part B Individual percentages of oil (oil%) and levels of estrogens in breast ADT

ADT									
		Levels (fmol/g)					Ratios		
**No.**	**Oil%**	E2	E1	2-MeO-E1	E1-S	E1G	E2/E1	2-MeO-E1/E1	E1-S/E1
1	60	229	771	<100	89	<250	0.30	<0.13	0.12
2	81	184	1,415	328	617	>250	0.13	0.23	0.44
3	67	318	1,035	105	269	<250	0.31	0.10	0.26
4	73	916	13,993	2,205	5,278	>250	0.07	0.16	0.38
5	81	82	452	<35	49	<250	0.18	<0.08	0.11
6	72	73	776	<35	63	<250	0.09	<0.05	0.08
7	62	66	376	<35	51	<250	0.18	<0.09	0.14
8	63	157	563	<35	112	<250	0.28	<0.06	0.20
9	82	94	530	<35	60	<250	0.18	<0.07	0.11
10	92	1,226	2,472	225	257	<250	0.50	0.09	0.10
11	81	1,642	2,876	294	146	<250	0.57	0.10	0.05
12	71	830	1,344	396	318	>250	0.62	0.29	0.24
13	85	112	479	<35	<19	<250	0.24	<0.07	<0.04
14	86	67	340	<35	<19	<250	0.20	<0.10	<0.06
15	84	1,515	2,869	910	250	>250	0.53	0.32	0.09
16	75	50	486	<35	50	<250	0.10	<0.07	0.10
17	94	3,075	4,567	484	355	<250	0.67	0.11	0.08
18	88	134	556	<35	48	<250	0.24	<0.06	0.09
19	72	107	578	<35	81	<250	0.19	<0.06	0.14
20	86	291	695	<100	70	<250	0.42	<0.14	0.10
21	82	96	627	<35	<35	<250	0.15	<0.06	<0.06
22	96	1,509	2,156	<100	200	<250	0.70	<0.05	0.09
23	97	478	1,297	<35	67	<250	0.37	<0.03	0.05
24	86	60	368	<35	<35	<250	0.16	<0.10	<0.10
25	84	455	1,554	156	276	>250	0.29	0.10	0.18
26	69	1,223	1,710	349	757	<250	0.72	0.20	0.44
27	75	<40	253	<35	70	<250	<0.16	<0.14	0.28
28	88	41	404	<35	42	<250	0.10	<0.09	0.10
29	79	292	1,444	116	94	<250	0.20	0.08	0.07
30	86	58	807	142	40	<250	0.07	0.18	0.05
31	84	809	3,708	131	104	<250	0.22	0.04	0.03
32	87	1,237	2,809	388	403	>250	0.44	0.14	0.14
33	80	201	660	<100	122	<250	0.30	<0.15	0.18
34	87	581	1,292	<100	<35	<250	0.45	<0.08	<0.03
35	88	419	1,051	130	46	<250	0.40	0.12	0.04
36	85	147	755	<35	35	<250	0.19	<0.05	0.05
37	91	190	783	<35	54	<250	0.24	<0.04	0.07
38	73	<40	249	<35	<35	<250	<0.16	<0.14	<0.14
39	83	421	861	<35	37	<250	0.49	<0.04	0.04
40	89	97	325	<35	69	<250	0.30	<0.11	0.21
41	93	1,081	2,536	106	195	<250	0.43	0.04	0.08
42	89	1,726	3,454	356	180	<250	0,50	0.10	0.05
43	90	99	602	<35	<19	<250	0.16	<0.06	<0.03
44	93	177	2,121	<35	<19	<250	0.08	<0.02	<0.01
45	85	613	1,560	<35	93	<250	0.39	<0.02	0.06
46	93	164	513	<35	<19	<250	0.32	<0.07	<0.04
47	91	45	438	<35	<19	<250	0.10	<0.08	<0.04

Part C Individual ratios of levels of estrogens in breast ADT to levels of estrogens in breast GLT (ADT/GLT). Grey color indicates ADT/GLT between 0.5 and 2, blue colors ADT/GLT <0.5 and red colors ADT/GLT >2. *, levels were <LOQ in ADT and <LOD in GLT; #, levels were <LOD in both tissues; </>, calculated with LOD/LOQ in one tissue, thus the calculated value represents the maximum (E1-S) and minimum (E2, 2-MeO-E1) and can be assumed to be even lower (E1-S) and higher (E2, 2-MeO-E1), respectively

	ADT/GLT			
**No.**	E2	E1	E1-S	2-MeO-E1
1	1.0	2.2	0.3	*>1.0
2	2.0	1.5	0.3	>3.3
3	1.6	1.9	0.5	>3.0
4	3.3	2.7	0.2	3.5
5	1.9	4.8	0.1	#
6	1.5	2.0	0.2	#
7	0.5	1.6	0.3	#
8	1.9	1.3	0.2	#
9	0.9	2.4	0.1	#
10	2.4	3.5	0.1	>6.4
11	1.7	1.5	0.1	1.0
12	1.2	1.7	0.2	>11.3
13	>2.8	4.2	<0.5	#
14	1.4	4.4	<0.1	#
15	1.5	2.4	0.2	2.4
16	>2.9	1.8	0.2	#
17	6.6	4.8	0.1	>13.8
18	2.8	5.9	0.2	#
19	2.1	2.5	0.3	#
20	2.2	2.4	0.1	*>1.0
21	1.6	1.4	<0.2	#
22	2.1	2.8	0.2	*>1.0
23	2.5	2.4	0.1	#
24	1.0	6.5	<0.1	#
25	2.2	3.8	0.1	>4.5
26	1.1	1.9	0.4	3.0
27	*>1.0	2.6	0.3	#
28	0.4	1.0	0.2	#
29	1.0	1.4	0.4	0.7
30	0.5	3.3	0.1	>4.1
31	2.3	3.7	0.1	>3.7
32	1.4	2.4	0.3	3.1
33	1.6	2.6	0.1	*>1.0
34	2.9	2.5	<0.2	*>1.0
35	3.0	5.8	0.1	>3.7
36	1.5	3.0	0.2	#
37	1.9	1.5	0.3	#
38	#	1.0	<0.4	#
39	6.1	5.6	0.2	#
40	0.8	2.0	1.1	#
41	3.0	3.6	0.1	>3.0
42	2.9	4.3	0.1	>10.2
43	1.4	6.3	<0.1	#
44	0.9	10.6	<0.1	#
45	1.2	2.5	0.2	#
46	2.4	5.2	<0.1	#
47	0.9	7.1	<0.1	#
