# Supplementary material for: Influence of breast cancer risk factors and intramammary biotransformation on estrogen homeostasis in the human breast
Source: Arch Toxicol. 2020 Jun 22;94(9):3013–25. doi: 10.1007/s00204-020-02807-1 (PMC7415756; doi:10.1007/s00204-020-02807-1)
Supplement: Supplementary file 3 — Supplementary file3 (PDF 416 kb) [file 204_2020_2807_MOESM3_ESM.pdf]

Archives of Toxicology

Influence of breast cancer risk factors and intramammary biotransformation on estrogen homeostasis in the human breast

Daniela Pemp, Leo N. Geppert, Claudia Wigmann, Carolin Kleider, René Hauptstein, Katja Schmalbach, Katja Ickstadt, Harald L. Esch, Leane Lehmann*

***Corresponding author:**
Prof. Dr. Leane Lehmann, Chair of Food Chemistry, University of Würzburg, Am Hubland, D-97074 Würzburg, Germany. Phone: +49 931 318-5481. Email: leane.lehmann@uni-wuerzburg.de.

Online Resource 3A. Heatmap of individual transcript levels in relation to the housekeeping gene HPRT1 in breast glandular tissue.

	Metabolizing enzymes																
		CYP				*HSD17B*				*SULT*				*UGT*			
No.	*COMT*	*1A1*	*1B1*	*19A1*	*GUSB*	*1*	*2*	*NQO1*	*STS*	*1A1*	*1A2*	*1A3/4*	*1E1*	*1A1*	*1A3/4*	*1A9*	*1A10*
1	0,58035	0,00096	0,40304	0,00144	1,10650	0,00622	0,29792	2,23268	0,49931	0,00549	0,01830	0,06134	0,00305	<LOD	<LOD	0,00011	0,00021
2	0,47100	0,04380	0,96393	0,00528	3,04829	0,00151	0,36148	3,93826	0,42632	0,00529	0,01160	0,08627	0,00172	0,00008	<LOD	0,00023	0,00024
3	0,37700	0,02010	0,57236	0,00143	1,27987	0,00348	0,23037	2,32000	0,36450	0,00401	0,00910	0,07433	0,00163	0,00006	<LOQ	0,00010	0,00015
4	0,22300	0,00124	3,09728	0,00400	1,01326	0,00110	0,21628	1,81541	0,38770	0,00393	0,01386	0,11391	<LOQ	0,00059	<LOD	0,00104	0,00168
5	0,55800	0,00011	1,05263	0,00568	1,67133	0,02272	0,33217	2,71401	0,59584	0,03612	0,05909	0,19238	0,00010	0,00015	<LOD	0,00120	0,00071
6	0,55400	0,00419	0,78241	0,00881	2,60810	0,00390	0,25279	4,88000	0,62894	0,00402	0,01193	0,09889	0,00190	0,00008	0,00010	0,00010	0,00034
7	0,41000	0,00225	0,60794	0,00650	1,44894	0,01507	0,32086	2,85000	0,67689	0,00707	0,01550	0,06342	0,00004	<LOQ	<LOQ	0,00019	0,00037
8	0,69112	0,00060	0,92787	0,00277	1,64947	0,01492	0,12175	1,34000	0,56880	0,01015	0,03050	0,21154	0,00103	<LOD	<LOD	0,00034	0,00012
9	3,73000	0,00296	2,02090	0,00658	1,24229	0,00656	0,05194	3,45402	2,32785	0,00285	0,02736	0,38400	0,00067	0,00066	0,00029	0,00014	0,00021
10	0,82100	0,00318	1,54384	0,00777	2,26098	0,00132	0,76663	3,50000	1,31000	0,01149	0,01640	0,65726	0,00196	<LOD	<LOD	0,00012	0,00040
11	0,77100	0,00003	0,83625	0,00039	0,85857	0,00099	0,14783	1,14000	0,26080	0,00359	0,00692	0,04860	0,00169	<LOD	<LOD	0,00004	0,00048
12	0,48300	0,00124	0,67971	0,00288	1,18838	0,00063	0,27624	1,58000	0,58479	0,00362	0,01025	0,06887	0,00014	0,00009	<LOD	0,00016	0,00009
13	1,78633	0,00096	0,95396	0,00092	1,82134	0,00046	0,30355	1,97000	0,44350	0,00406	0,02781	0,11574	0,00047	0,00004	<LOD	0,00028	0,00033
14	0,19683	0,00035	3,13399	0,00077	0,92466	0,00193	0,20791	1,98083	0,11500	0,00451	0,17776	0,14968	0,00121	<LOD	<LOD	0,00020	0,00082
15	1,09000	0,04290	0,87418	0,00373	2,01670	0,02018	0,14711	6,09276	0,78731	0,01360	0,03879	0,13416	0,00004	0,00023	<LOD	0,00008	0,00012
16	0,62503	0,00089	1,41716	0,01518	1,34164	0,00049	0,15156	1,94000	2,19314	0,00466	0,00793	0,09280	0,00039	<LOD	<LOD	0,00013	<LOD
17	0,43200	0,00063	0,99585	0,00445	1,32134	0,00087	0,30020	0,58640	1,08749	0,00335	0,01120	0,24065	0,00020	0,00044	<LOD	0,00022	0,00017
18	1,13000	0,00012	0,37919	0,00052	0,95198	0,00269	0,05880	3,68000	0,27319	0,00572	0,00725	0,18276	0,00179	0,00028	<LOD	0,00029	0,00014
19	5,95046	0,01950	2,37347	0,00088	1,24747	0,00433	0,36198	1,39785	11,70457	0,00280	0,00594	0,52778	0,00002	0,00030	<LOD	0,00034	0,00036
20	0,57197	0,11400	0,69544	0,00097	1,90660	0,00351	0,41783	1,92242	0,75367	0,00220	0,01830	0,04058	0,00004	0,00012	<LOD	0,00051	0,00065
21	0,77200	0,01750	0,87721	0,00213	3,40818	0,00477	0,23374	4,87025	0,96393	0,00482	0,00738	0,08432	0,03382	0,00060	<LOD	0,00118	0,00166
22	0,79443	0,00051	2,18102	0,00200	2,08782	0,00041	0,25331	2,13059	1,17283	0,00319	0,02012	0,07290	0,00068	<LOD	<LOD	0,00012	0,00011
23	0,79200	0,00042	2,15248	0,01324	2,29899	0,00129	0,11447	1,22536	0,62028	0,00341	0,00872	0,10912	0,00500	0,00038	<LOQ	0,00023	0,00048
24	1,03097	0,00183	0,45597	0,00115	2,20993	0,00068	0,38236	2,79000	0,38800	0,00319	0,01601	0,16188	0,00272	0,00030	<LOD	0,00032	0,00028
25	0,28900	0,00003	0,72598	0,00540	1,02101	0,00053	0,06556	1,23000	0,35824	0,00222	0,00430	0,32376	0,00169	0,00005	<LOD	0,00010	0,00010
26	0,52600	0,00133	1,30044	0,00044	1,15349	0,00020	0,41123	0,71100	0,33798	0,00276	0,00472	0,06916	0,00003	<LOD	<LOD	0,00013	0,00036
27	0,77700	0,00136	1,38407	0,00213	1,05991	0,00071	0,58524	1,31670	0,67050	0,00379	0,00771	0,08219	0,00004	0,00009	<LOD	0,00012	0,00010
28	1,06000	0,00075	1,59549	0,01113	3,20872	0,00317	1,46713	4,46000	0,68113	0,00397	0,02120	0,09652	0,00090	0,00055	<LOQ	0,00048	0,00076
29	0,78200	0,00007	0,44751	0,00115	1,07549	0,00075	0,24759	1,33000	0,28600	0,00460	0,00570	0,23277	0,00095	0,00006	<LOD	0,00020	0,00009
30	0,86600	0,02435	2,30537	0,00255	1,77645	0,00034	0,20278	1,00204	0,49896	0,00237	0,01081	0,08340	0,00005	<LOD	<LOD	0,00003	0,00018
31	0,89900	0,00073	0,20863	0,00180	2,52801	0,00682	0,35282	2,64000	0,36900	0,00247	0,03930	0,25402	0,00237	<LOD	<LOD	0,00019	<LOD
32	0,71698	0,00410	1,09885	0,00957	2,31338	0,01061	0,64618	2,56327	1,35000	0,00565	0,01253	0,12500	0,00012	<LOD	<LOD	0,00007	0,00004
33	0,45125	0,00165	1,12350	0,00154	1,20748	0,00389	0,15921	3,52139	0,47041	0,00418	0,01115	0,09903	0,00048	<LOD	<LOD	<LOD	<LOD
35	0,61000	0,00247	0,80329	0,00105	1,60659	0,00716	0,49243	1,72000	0,91828	0,00255	0,00889	0,07270	0,00005	<LOD	<LOQ	<LOD	0,00015
36	0,60249	0,01286	0,32331	0,00300	4,75683	0,00058	0,31997	4,11107	0,59378	0,00160	0,00839	0,04704	0,00178	0,00037	<LOQ	0,00039	0,00066
38	0,71600	0,34200	1,00626	0,00555	1,73748	0,01414	0,45188	2,27000	0,55633	0,00851	0,01360	0,23619	0,00024	<LOD	<LOD	0,00015	0,00037
39	0,46200	0,00002	0,60584	0,00078	0,87297	0,00010	0,75471	0,24300	0,16700	0,00201	0,00252	0,06769	0,00003	0,00019	<LOD	0,00011	0,00019
40	0,56900	<LOD	1,17772	0,00889	1,85318	0,02057	0,10366	3,61000	0,52400	0,00354	0,01750	0,13351	0,00057	0,00007	0,00006	<LOD	<LOD
41	1,26400	0,00011	1,65061	0,00574	1,61217	0,00386	0,21991	2,66000	0,22700	0,00169	0,01246	0,09300	0,00036	<LOD	0,00043	<LOQ	0,00002
42	1,18000	0,00054	1,78757	0,00126	1,26400	0,00123	0,91257	1,33000	0,56600	0,00584	0,01064	0,15700	0,00154	<LOD	<LOD	0,00001	0,00006
43	0,80200	0,00081	1,36415	0,00509	1,43296	0,00458	0,48397	1,84724	0,32900	0,00444	0,00629	0,10006	0,00024	<LOD	<LOD	<LOD	<LOD
44	0,96326	0,18492	2,24233	0,00026	1,67365	0,00351	0,06731	3,06162	0,41639	0,00387	0,00920	0,13660	0,02272	<LOD	<LOD	0,00003	<LOD
45	1,63580	0,00040	1,44894	0,01250	1,72788	0,00155	0,19833	1,90495	0,63156	0,00513	0,02339	0,07081	0,00007	n.d.	<LOQ	0,00003	0,00003
46	1,16949	0,00082	1,49542	0,00561	3,05945	0,00042	0,40475	5,39057	0,61833	0,00632	0,01103	0,07167	0,00046	n.d.	<LOD	<LOQ	0,00007
47	0,87055	0,00108	8,32817	0,00329	1,78881	0,00331	0,13149	2,58999	0,38236	0,00266	0,01800	0,08339	0,00057	n.d.	<LOQ	<LOQ	0,00009

Legend

- <LOD
- <LOQ
- ≤ 0.001
- >0.001 to ≤ 0.010
- >0.010 to ≤ 0.100
- > 0.100 to ≤ 1.000
- >1.000 to ≤ 10.000
- >10.000 to ≤ 100.000

Note:
No transcript levels were determined in specimen number (No.) 34 and 37.

Archives of Toxicology

Influence of breast cancer risk factors and intramammary biotransformation on estrogen homeostasis in the human breast

Daniela Pemp, Leo N. Geppert, Claudia Wigmann, Carolin Kleider, René Hauptstein, Katja Schmalbach, Katja Ickstadt, Harald L. Esch, Leane Lehmann*

***Corresponding author:**
Prof. Dr. Leane Lehmann, Chair of Food Chemistry, University of Würzburg, Am Hubland, D-97074 Würzburg, Germany. Phone: +49 931 318-5481. Email: leane.lehmann@uni-wuerzburg.de.

Online Resource 3B. Heatmap of individual transcript levels in relation to the housekeeping gene HPRT1 in breast adipose tissue.

	Metabolizing enzymes																	Transcription factors						
		CYP				HSD17B				SULT				UGT						ESR			NR	
No.	*COMT*	*1A1*	*1B1*	*19A1*	*GUSB*	*1*	*2*	*NQO1*	*STS*	*1A1*	*1A2*	*1A3/4*	*1E1*	*1A1*	*1A3/4*	*1A9*	*1A10*	*AHR*	*ARNT*	*1*	*2*	*NFE2L2*	*1I2*	*1I3*
1	1,30405	0,00130	1,62450	0,04636	4,77996	0,02050	0,06882	18,55446	2,23767	0,00878	0,06580	0,08778	<LOD	0,00068	0,00128	0,00012	0,00008	6,95958	3,02933	1,95000	0,12336	4,56622	0,00595	<LOD
2	0,73458	0,17182	1,12350	0,02264	2,44698	0,01053	0,17900	24,13748	6,69925	0,00389	0,03080	0,17458	0,00038	0,00079	0,01986	0,00009	0,00035	4,00000	1,27987	13,36141	0,06020	2,80305	0,00175	<LOD
3	0,63949	0,16021	0,29504	0,01274	2,08638	0,04400	0,15669	9,76118	44,47738	0,00314	0,01028	0,11891	0,00046	0,00326	0,00330	0,00025	0,00042	3,18215	3,44618	6,17740	0,17641	3,54307	0,00415	0,00020
4	0,19144	0,00057	0,95132	0,00667	2,18102	0,04856	0,22000	20,51808	1,29954	0,01819	0,05900	0,12780	0,00002	0,00119	0,01172	0,00022	0,00037	4,53468	2,02230	1,00000	0,03031	6,89715	0,01320	0,00024
5	0,60710	0,00099	2,19162	0,00332	1,93724	0,02008	0,04730	11,04000	4,31093	0,00803	0,02910	0,15800	0,00012	0,00023	0,00034	0,00027	0,00059	2,65921	4,00833	1,43000	0,07889	2,96493	0,00286	0,00005
6	1,02883	0,00069	0,60921	0,06475	1,92119	0,02107	0,03250	15,32468	2,51577	0,00709	0,02530	0,08213	0,00014	0,00072	0,02642	0,00006	<LOD	4,71089	2,83825	2,87388	0,05172	4,54727	0,00643	<LOD
7	0,51799	0,00474	0,72146	0,02160	2,34892	0,02190	0,07200	20,43650	6,24629	0,01620	0,03780	0,10852	0,00023	0,00072	0,00058	0,00019	0,00096	5,95871	3,94220	1,44000	0,11639	5,79175	0,00196	0,00034
8	0,56370	0,00043	0,53737	0,00934	1,95748	0,04356	0,21500	7,28350	1,12194	0,00786	0,02440	0,26720	<LOD	0,00012	0,00041	<LOD	<LOD	4,87026	3,14487	3,23000	0,10195	3,27161	0,00787	<LOD
9	1,16878	<LOQ	1,09202	0,07562	2,68328	0,04969	0,03780	20,70534	1,82893	0,01094	0,06263	0,09612	0,00038	0,00096	0,00471	0,00034	0,00020	6,92589	3,16455	1,22000	0,07140	12,99604	0,00393	0,00007
10	1,10957	0,02204	1,13524	0,00649	30,44218	0,00761	1,25000	82,73801	1,39959	0,00900	0,01250	0,12260	0,00149	0,00286	0,00010	0,00183	0,00039	4,52526	1,62902	1,48000	0,01263	15,21055	0,00488	0,00007
11	0,85500	0,00029	0,94868	0,03492	2,04627	0,02294	0,13200	21,75075	2,26891	0,01282	0,08556	0,13131	<LOD	<LOD	0,00010	0,00004	0,06498	5,71598	2,93223	0,76844	0,02814	3,66040	0,00526	0,00010
12	0,65747	0,00122	0,83683	0,00906	1,95071	0,02250	0,21900	9,74338	1,97383	0,00665	0,03472	0,24656	0,00010	0,00012	0,00036	0,00007	<LOD	6,77396	2,41664	1,82000	0,04050	4,00277	0,01185	0,00018
13	0,82645	0,00074	0,70466	0,01303	2,94445	0,08440	0,07270	17,07004	3,76330	0,01490	0,23300	0,18855	<LOD	0,00931	0,04190	<LOQ	0,00058	6,64837	4,63638	1,40000	0,22531	7,89000	0,00330	<LOD
14	0,86214	0,00223	0,52961	0,01255	2,24389	0,01130	0,00844	29,54160	4,83998	0,02260	0,13800	0,16369	<LOD	<LOD	<LOQ	0,00008	0,00042	4,25453	2,88586	0,72900	0,12032	5,52510	0,00498	0,00005
15	0,70760	0,03044	0,60878	0,00648	2,22222	0,03356	0,13000	22,69999	2,22068	0,01260	0,07580	0,13927	<LOQ	<LOQ	0,00075	<LOQ	<LOD	3,90413	2,59728	1,30000	0,06493	6,72251	0,00246	0,00009
16	1,37268	0,00334	1,30858	0,01328	3,11666	0,06588	0,21900	20,71703	34,10710	0,01495	0,08833	0,26665	0,00017	<LOD	0,00118	0,00043	0,00046	14,24121	4,09255	3,43426	0,32601	8,62784	0,00696	<LOD
17	1,04319	0,00588	0,81000	0,01112	1,96973	0,04129	0,23100	16,03297	3,78948	0,01289	0,08230	0,08414	0,00012	0,00454	0,00413	0,00009	0,00067	4,24864	2,75681	0,58927	0,06372	2,75681	0,00682	0,00027
18	0,74072	0,00101	0,37010	0,00886	2,28311	0,05000	0,00512	23,69394	3,19541	0,01040	0,10200	0,18225	0,00010	0,00052	0,00177	0,00009	0,00074	4,57890	3,57266	1,19000	0,13076	3,21763	0,00270	0,00015
19	0,73255	0,97333	2,60088	0,00430	3,33341	0,06550	0,12100	9,53290	1,68529	0,01498	0,03106	0,09434	<LOD	0,00010	0,00009	0,00006	0,00025	9,36968	3,48220	1,73748	0,15000	5,38520	0,00588	<LOD
20	0,74278	0,91828	0,60123	0,00911	2,34080	0,03315	0,02920	17,58930	5,94633	0,01746	0,09910	0,22893	0,00004	0,00055	0,00035	0,00015	0,00044	5,31843	3,14051	2,97000	0,14409	5,56353	0,00357	0,00014
21	1,11497	0,00323	0,41928	0,01354	2,95878	0,09830	0,13400	15,10851	2,59368	0,00557	0,03863	0,16278	0,00016	0,00171	0,01908	0,00008	0,00026	4,76013	3,56771	1,92786	0,18999	4,03901	0,00606	<LOD
22	3,12315	0,00116	1,59218	0,02753	3,26708	0,20700	0,12500	23,57608	3,07375	0,01512	0,07970	0,33079	<LOD	0,00188	0,00260	0,00010	0,00023	10,45412	3,74249	1,71000	0,09434	7,47462	0,00411	0,00015
23	0,73002	0,00075	0,77004	0,01363	2,41664	0,03986	0,01890	18,77936	2,37019	0,00321	0,01730	0,16632	<LOQ	0,00030	0,00053	0,00005	0,00041	5,56353	3,81055	1,63000	0,09062	3,34499	0,00194	0,00005
24	1,03526	0,00126	0,69303	0,02930	1,79502	0,12141	0,16400	15,69333	4,11531	0,00527	0,02090	0,10980	0,00019	0,00109	0,00255	0,00006	0,00025	4,23687	1,45297	1,76908	0,13660	2,16295	0,00597	0,00002
25	0,18301	0,00048	0,27548	0,00438	1,40834	0,02710	0,06450	20,85631	1,95477	0,00951	0,02950	0,19820	0,00060	0,00074	0,00087	0,00009	0,00021	3,68586	2,20993	1,66000	0,09559	4,96571	0,00304	0,00011
26	11,07320	0,03182	10,77040	0,01045	2,52801	0,03890	0,14100	9,01890	140,94612	0,00883	0,12800	0,43287	0,00006	0,00721	0,01156	0,00012	0,00060	3,96962	4,20761	29,30000	0,11801	11,03489	0,01389	<LOD
27	1,50629	0,00973	4,71089	0,00803	2,49666	0,03640	1,79000	8,28195	2,35545	0,00663	0,04290	0,93239	0,00007	0,00123	0,00546	<LOQ	0,00012	10,60006	5,32950	6,70000	0,12294	4,51273	0,01351	0,00005
28	4,78660	0,00180	0,94475	0,03506	4,01946	0,15600	0,13900	12,67152	5,26342	0,01839	0,04860	0,17690	0,00003	0,00048	0,00338	0,00017	<LOD	5,15153	4,01111	5,27437	0,21110	4,54727	0,00184	<LOD
29	1,25093	0,00078	0,63861	0,01214	1,97657	0,23500	0,05160	9,45881	1,55833	0,01579	0,01340	0,06529	0,00007	0,00033	0,00029	0,00007	0,00044	3,03774	4,20469	1,92519	0,13416	2,14652	0,00203	0,00011
30	1,94666	0,11502	1,46815	0,00330	2,27206	0,02230	0,21500	9,16582	1,96156	0,00960	0,05052	0,13966	0,00003	0,00020	0,00004	0,00017	0,00058	5,09824	2,66660	1,47000	0,04766	4,84333	0,01055	0,00012
31	0,98487	0,00074	1,49174	0,03766	2,48630	0,06600	0,69100	17,76225	2,30378	0,01290	0,05060	0,14339	0,00008	0,00056	0,00032	0,00053	0,00068	5,71202	1,49174	1,16000	0,03742	3,65000	0,00552	0,00007
32	0,75944	0,00242	0,38985	0,01047	1,83274	0,01856	0,00462	24,10716	3,70635	0,01145	0,03510	0,12234	<LOD	<LOD	<LOD	0,00005	0,00013	6,34667	5,94221	0,63244	0,11494	3,11234	0,00230	0,00004
33	0,53477	0,00076	0,33986	0,00675	1,72190	0,01978	0,05840	14,58851	3,54307	0,00411	0,05115	0,11891	0,00030	0,00049	0,00097	0,00011	0,00013	5,37774	2,72263	1,06955	0,07716	3,04196	0,00798	0,00025
35	2,07484	0,00218	0,53292	0,01733	2,21914	0,01369	0,10600	21,36056	13,71802	0,03169	0,11000	0,20349	0,00007	0,00031	0,00587	0,00006	0,00027	6,60702	4,72725	2,04000	0,11118	9,48074	0,00640	<LOD
36	1,55079	0,07955	2,06051	0,01483	1,91986	0,08981	0,01860	9,05226	1,94936	0,00676	0,02010	0,12905	0,00016	0,00024	0,00007	<LOQ	<LOD	4,17854	2,80305	1,46612	0,04928	5,09000	0,00494	<LOD
37	5,69225	0,86634	0,98966	0,01699	3,78685	0,07410	0,01070	47,56503	3,15142	0,02357	0,17000	0,26371	0,00015	0,00042	0,00036	0,00053	0,00021	6,47105	4,30795	3,83440	0,15756	6,44866	0,00273	<LOQ
38	0,54450	1,60214	1,39571	0,00597	3,11018	0,05150	0,08290	11,35995	2,53678	0,02437	0,04500	0,18238	<LOD	0,00024	0,00228	<LOD	<LOD	6,89237	4,11816	2,48000	0,23521	13,90000	0,00872	0,00027
39	1,14631	0,00119	0,31842	0,03342	2,29262	0,02722	0,00897	10,79958	2,72452	0,00625	0,01460	0,11777	<LOD	0,00068	0,00118	0,00013	0,00027	3,59502	3,18656	0,89503	0,09195	5,82396	0,00198	0,00004
40	0,73918	0,00023	1,04826	0,01416	2,66106	0,09470	0,14100	18,73050	4,19596	0,01366	0,04095	0,30062	0,00022	0,00018	0,00041	0,00009	<LOD	5,69620	2,93834	1,88296	0,14824	9,59312	0,00693	0,00014
41	0,87418	<LOQ	0,35014	0,02273	3,21986	0,09620	0,00895	35,63953	1,70055	0,02484	0,03590	0,23504	<LOQ	0,00589	0,01200	0,00032	0,00077	4,29602	2,60991	0,45300	0,09993	9,01920	0,00472	0,00015
42	1,17528	0,00099	1,21672	0,01106	2,90392	0,12260	0,48500	17,44135	5,04551	0,01423	0,05540	0,15347	0,00009	0,00018	0,00043	<LOQ	<LOD	6,69925	3,26255	1,82000	0,09679	9,58647	0,01200	<LOD
43	0,18112	0,00030	0,92402	0,00098	0,47369	0,00773	0,07860	8,93622	0,52486	0,00109	0,00611	0,88884	0,00010	0,00077	0,00103	0,00001	0,00016	1,35849	0,82074	10,02132	0,01373	1,80000	0,00245	<LOD
44	1,02527	0,41009	1,53262	0,06944	1,51677	0,01636	0,00106	8,54788	1,12272	0,00587	0,02432	0,09356	<LOD	0,00029	0,00227	0,00034	0,00072	5,55967	1,64148	0,61685	0,01705	3,72696	0,00154	0,00018
45	1,31859	0,00071	10,07006	0,03834	2,55443	0,03340	0,03850	18,15800	5,01761	0,00330	0,04591	0,26720	<LOQ	<LOD	0,00022	<LOQ	0,00009	3,95588	3,12965	1,17446	0,05042	3,53571	0,00456	<LOD
46	0,58398	0,00363	2,01670	0,01379	3,06312	0,06177	0,02172	12,02750	1,61552	0,01260	0,12509	0,13112	0,00181	0,00007	0,00007	<LOQ	0,00007	8,88271	4,43520	2,39662	0,03986	5,23069	0,00781	0,00010
47	0,82302	0,00097	0,95595	0,03197	2,09507	0,03110	0,06806	9,30070	1,93053	0,00620	0,04671	0,16887	0,00011	0,00091	0,00474	<LOQ	0,00018	4,55989	4,03341	2,45888	0,06873	5,40015	0,00400	<LOD

Legend

- <LOD
- <LOQ
- ≤ 0.001
- >0.001 to ≤ (
- >0.010 to ≤ (
- > 0.100 to ≤
- >1.000 to ≤ 1
- >10.000 to ≤

Note:
No transcript levels were determined in specimen number (No.) 34.
