# Supplementary material for: Influence of breast cancer risk factors and intramammary biotransformation on estrogen homeostasis in the human breast
Source: Arch Toxicol. 2020 Jun 22;94(9):3013–25. doi: 10.1007/s00204-020-02807-1 (PMC7415756; doi:10.1007/s00204-020-02807-1)
Supplement: Supplementary file 4 — Supplementary file4 (PDF 828 kb) [file 204_2020_2807_MOESM4_ESM.pdf]

Archives of Toxicology

Influence of breast cancer risk factors and intramammary biotransformation on estrogen homeostasis in the human breast

Daniela Pemp, Leo N. Geppert, Claudia Wigmann, Carolin Kleider, René Hauptstein, Katja Schmalbach, Katja Ickstadt, Harald L. Esch, Leane Lehmann*

***Corresponding author:**
Prof. Dr. Leane Lehmann, Chair of Food Chemistry, University of Würzburg, Am Hubland, D-97074 Würzburg, Germany. Phone: +49 931 318-5481.
Email: leane.lehmann@uni-wuerzburg.de.

Online Resource 4. Setup, outcome and interpretation of multiple linear regression models using stepwise forward selection.

Multiple linear regression models using stepwise forward selection were applied to assess up to 32 explanatory variables (exVARs, continuous as well as categorical) possibly influencing levels of estrogens, ratios thereof, mass percentage of oil (oil%) in breast glandular (GLT) and adipose (ADT) tissues as well as levels of transcripts encoding CYP19A1, GUSB and HSD17B2 in breast ADT as dependent variables.

ExVARs tested were: Age (continuous), menopausal status (categorical, reference group premenopausal, and further groups perimenopausal, periMP, and postmenopausal, postMP), lobule type (categorial, reference group lobule type 2/3, Lob2/3, and further groups lobule type 1 nulliparous, Lob1np, and lobule type 1 parous, Lob1p. To reflect continuous influence of age as well as abrupt influence of menopausal status on dependent variables, both the potential exVARs age and menopause (Fig. 2), were included into the models. Further exVARs tested were oil% in GLT and ADT, respectively (continuous), BMI (continuous), smoking habits (binary, reference group non-smoker), intake of estrogen active drugs (EAD, categorial, reference group no intake of EAD and further groups intake of ethinyl estradiol, EE, and intake of 17β-estradiol, E2,-releasing drugs, ERD).

In models with estrogen levels as dependent variables, further exVARs tested were tissue levels of the direct precursor estrogen(s) and/or direct biotransformation product(s) of the dependent variable and levels of transcripts encoding enzymes directly forming or further metabolizing the dependent variable according to Fig. 1. In models with ratios of levels of different estrogens as dependent variables, further exVAR considered were levels of transcripts encoding enzymes directly forming or further metabolizing at least one of the estrogens involved in the ratio. In models with oil% in GLT or ADT as dependent variable, levels of E2 putatively influencing oil% by interaction with lipid metabolism, and GLT characterized by large area of adipocytes (laaGLT, only in GLT, reference group saaGLT) were further considered as exVARs. In models with levels of transcripts as dependent variable, levels of transcripts encoding transcription factors known to regulate genes encoding enzymes involved in biotransformation were further considered as exVARs.

Mosaic plots characterizing the study population regarding categorical variables are depicted in Online Resource 8.

If levels of estrogens or transcripts were below limit of quantification (LOQ) in >40% of samples (Online Resources 2 and 3, respectively), they were not included as exVARs.

For levels of transcripts or estrogens below the respective limit of detection (<LOD)/<LOQ), LOD/LOQ was set, respectively. When levels of transcripts or estrogens were <LOQ in >1 sample and ≤40% of samples, the levels of the respective transcript/estrogen were included as the qualitative presence of the exVAR (binary exVARq, compared to levels <LOQ) in addition to the continuous exVARs.

If in the computed model observations with Cook's Distance >1 appeared, they were removed and the model was computed anew. This process was repeated until no conspicuous observations (CO) remained. To achieve normal distribution, dependent variables were logarithmized. Data distributions were evaluated in Quantile-Quantile plots with simulated confidence bands. Constant standard deviations of the errors were evaluated using scale-location plots. To check the model assumption of independent identically distributed errors, the residual vs. fitted values plot was used. For every final model, adjusted coefficient of determination (R^2), the numbers of CO removed, the number of observations contributing to the final model (n, maximum 45 due to two specimen without information on EAD), and the ratio of observations per exVAR (O/exVAR) is given. For each exVAR selected, the regression coefficient (which represent the mean changes in the dependent variables for one unit of change in the respective exVAR while holding other predictors in the models constant), their confidence interval (CI), as well as the *P* value were given.

Spearman's rank correlation analysis was performed to identify collinearity between numerical exVARs which might hinder each others selection and/or influence each others *P* values within the models. In the case of variables with > 1 level below LOD or LOQ, correlation was calculated with randomly distributed ranks for ties 10,000 times and highest Spearman correlation coefficients (R) and lowest *P* values are given to rather overestimate collinearity. Relationship between categorical and numerical exVARs was evaluated by comparison of medians using unpaired Wilcoxon tests. Correlations and relationships between exVARs with *P* values < 0.001 were listed below the table of each model with the respective *P* value. If such exVARs were selected for the final model, a comment addressing possible consequences was added.

Associations of exVARs selected and exhibiting *P* values ≤ 0.25 are interpreted in the last column of each table.

E1, estrone; E1-S, E1-sulfate; 2-MeO-E1, 2-methoxy-E1; +/-, positive/negative association; ↑, increase; ↓, decrease; exVAR colored black, selected, *P* values < 0.05; exVAR colored blue, selected, 0.10 < P value ≥ 0.05; exVAR colored light-blue, selected, 0.25 < *P* value ≥ 0.10; exVAR coloured grey, selected, *P* values > 0.25.

E2 in GLT
CO = 0; n = 43; O/exVAR = 14.3; R^2 = 0.65

exVAR (% <LOQ)	Selected in model	Regression Coefficient	CI 2.5%	CI 97.5%	*P* Value	Interpretation
Age[1]						
Menopause						
Lobule type						
Oil%						
BMI	BMI	+ 0.0430	- 0.0071	+ 0.0932	0.09	Body adipose tissue (including ADT)↑ → E2 formation↑ → Diffusion into GLT↑
Smoking[2]						
EAD						
E1	E1	+ 0.0020	+ 0.0015	+ 0.0025	<0.001	Precursor of E2
CYP1A1[2] (2%)						
CYP1B1						
CYP19A1						
GUSB						
HSD17B1						
HSD17B2						
STS						
SULT1A1						
SULT1A2						
SULT1A3/4						
SULT1E1[1]						
UGT1A10 (14%)	*UGT1A10*	- 450.2	- 1107.1	+ 206.8	0.17	Enzyme catalyzes biotransformation of E2
UGT1A10q						

Note:
[1], Correlation of Age and *SULT1E* (GLT): R=0.51, *P* <0.001 (4.38×10^{-4}).
[2], Significant difference of *CYP1A1* levels (GLT) in smokers (higher) and non smokers (lower): *P* <0.001 (4.47×10^{-4}).

E2 in ADT

CO = 0; n = 44; O/exVAR = 5.5; $R^2 = 0.84$

**exVAR (% <LOQ)**	**Selected in model**	**Regression Coefficient**	**CI 2.5%**	**97.5%**	***P* Value**	**Interpretation**
Age	Age	- 0.0010	- 0.0267	+ 0.0059	0.20	Circulating E2↓ → diffusion into GLT↓ and/or intra-tissue production of E2↓
Menopause						
Lobule type	Lob1np	- 0.4816	- 0.9217	- 0.0414	0.033	No (simple) interpretation possible
	Lob1p	- 0.6945	- 1.1710	- 0.2179	0.006	No (simple) interpretation possible, collinearity periMP and postMP unlikely
Oil%						
BMI						
Smoking[1]						
EAD						
E1	E1	+ 0.0010	+ 0.0008	+ 0.0011	<0.001	Precursor of E2
CYP1A1[1] (5%)	*CYP1A1*	- 0.4912	- 1.0039	+ 0.0214	0.06	Enzyme catalyzes biotransformation of E2
CYP1A1q						
CYP1B1						
CYP19A1	*CYP19A1*	- 7.209	-16.5113	+ 2.0938	0.12	Downregulation by product E2
GUSB	*GUSB*	+ 0.1149	+ 0.0511	+ 0.1788	<0.001	Enzyme catalyzes formation of E2
HSD17B1						
HSD17B2	*HSD17B2*	- 2.262	- 3.6709	- 0.8534	0.002	Enzyme catalyzes biotransformation of E2
STS						
SULT1A1[2]						
SULT1A2[2]						
SULT1A3/4						
SULT1E1 (34%)						
SULT1E1q						
UGT1A1 (14%)[3]						
UGT1A1q						
UGT1A3/4 (5%)[3]						
UGT1A10 (20%)						
UGT1A10q						

Note:

[1], Significant difference of *CYP1A1* levels (ADT) in smokers (higher) and non smokers (lower): *P* <0.001 (*6*.03x10^{-5}).

[2], Correlation of *SULT1A1* (ADT) and *SULT1A2* (ADT): R=0.55, *P*<0.001 (1.12x10^{-4}).

[3], Correlation of *UGT1A3/4* (ADT) and *UGT1A1* (ADT): R=0.71, *P*<0.001 (3.32x10^{-7}).

E1 in GLT

CO = 0; n = 43; O/exVAR = 2.7; R^2 = 0.83

exVAR (% <LOQ)	Selected in model	Regression Coefficient		CI 2.5%		CI 97.5%	*P* Value	Interpretation
Age[1]								
Menopause	PeriMP	-	0.3282	- 0.7374	+	0.0809	0.11	Circulating E1↓ → diffusion into GLT↓ and/or intra-tissue production of E1↓
	PostMP	-	0.8604	- 1.3352	-	0.3856	<0.001	Circulating E1↓ → diffusion into GLT↓ and/or intra-tissue production of E1↓
Lobule type	Lob1np	+	0.2073	- 0.0194	+	0.6089	0.30	No interpretation due to *P* >0.25
	Lob1p	-	0.1355	- 0.5690	+	0.2979	0.53	No interpretation due to *P* >0.25
Oil%	Oil%	+	0.0218	+ 0.0103	+	0.0332	<0.001	Distribution oil/aqueous phase* or production in adipocytes
BMI	BMI	+	0.0392	+ 0.0035	+	0.0749	0.033	Body adipose tissue (including ADT)↑ → E1 production↑ → Diffusion into GLT↑
Smoking[2]	Smoking	+	0.2975	- 0.1058	+	0.7008	0.14	No (simple) interpretation possible
EAD	EE	-	0.5408	- 1.0765	-	0.0052	0.048	Circulating E1↓ → diffusion into GLT↓ and/or intra-tissue production of E1↓
	ERD	+	0.2929	- 0.4020	+	0.9878	0.39	No interpretation due to *P* >0.25
E2 (7%)[3]	E2	+	0.0013	+ 0.0007	+	0.0019	<0.001	Precursor of E1
E2q								
E1-S[3]	E1-S	+	0.0002	<0.0001	+	0.0003	0.027	Precursor of E1
E1-Gq	E1-Gq	+	0.0454	+ 0.4468	-	0.3560	0.82	No interpretation due to *P* >0.25
CYP1A1[2] (2%)								
CYP1B1								
GUSB	*GUSB*	+	0.1605	- 0.0375	+	0.3585	0.11	Enzyme catalyzes formation of E1
CYP19A1								
HSD17B1								
HSD17B2								
STS								
SULT1A1								
SULT1A2								
SULT1A3/4								
SULT1E1[1]	*SULT1E1*	+	13.26	-11.46		+37.99	0.28	No interpretation due to *P* >0.25
UGT1A9 (17%)[4]								
UGT1A9q	*UGT1A9q*	+	0.5088	+ 0.1020	+	0.9157	0.016	Upregulation by substrate E1
UGT1A10 (14%)[4]								
UGT1A10q	*UGT1A10q*	-	0.2813	- 0.7118	+	0.1491	0.19	Enzyme catalyzes biotransformation of E1

Note:

[1], Correlation of Age and *SULT1E1* (GLT): R=0.51, P<0.001 (4.38×10^{-4}).

[2], Significant difference of *CYP1A1* levels (GLT) in smokers (higher) and non smokers (lower): P <0.001 (4.47×10^{-4}).

[3], Correlation of E2 (GLT) and E1-S (GLT): R=0.65, P<0.001 (4.37×10^{-6}).

[4], Correlation of *UGT1A9* (GLT) and *UGT1A10* (GLT): R=0.69, P<0.001 (8.15×10^{-7}).

*Because of its lipophilicity E1 distributes preferentially into the oil phase.

Comments:

P values of the exVARs E2 and E1-S are possibly too high (see footnote[3]).

E1 in ADT

CO = 1; n = 43; O/exVAR = 2.9; R^2 = 0.84

exVAR (% <LOQ)	Selected in model	Regression Coefficient	CI 2.5%	CI 97.5%	*P* Value	Interpretation
Age	Age	- 0.0072	- 0.0170	+ 0.0026	0.15	Circulating E1↓ → diffusion into GLT↓ and/or intra-tissue production of E1↓
Menopause[1]						
Lobule type						
Oil%						
BMI	BMI	+ 0.0558	+ 0.0225	+ 0.0892	0.002	Body adipose tissue (including ADT)↑ → E1 production↑
Smoking[2]	Smoking	+ 0.3240	+ 0.0565	+ 0.5914	0.019	No (simple) interpretation possible
EAD						
E2[3]	E2	+ 0.0007	+ 0.0005	+ 0.0009	<0.001	Precursor of E1
E1-S[1,3] (21%)						
E1-Sq	E1-Sq	+ 0.2757	- 0.0397	+ 0.5912	0.084	Precursor of E1
E1Gq	E1Gq	+ 0.5721	+ 0.2322	+ 0.9120	0.002	Precursor of E1
CYP1A1[2] (5%)						
CYP1A1q	*CYP1A1q*	+ 0.6249	+ 0.0224	+ 1.2274	0.043	Upregulation by substrate E1
CYP1B1						
CYP19A1	*CYP19A1*	+ 12.35	+ 5.57	+19.12	<0.001	Enzyme catalyzes formation of E1
GUSB	*GUSB*	- 0.1689	- 0.3383	+ 0.0004	0.050	Downregulation by product E1
HSD17B1	*HSD17B1*	+ 1.914	- 0.4054	+ 4.2335	0.10	Upregulation by substrate E1
HSD17B2	*HSD17B2*	+ 1.045	- 0.0148	+ 2.0744	0.047	Enzyme catalyzes formation of E1
STS						
SULT1A1[4]						
SULT1A2[4]	*SULT1A2*	- 1.001	- 3.6736	+ 1.6723	0.45	No interpretation due to *P* >0.25
SULT1A3/4						
SULT1E1 (35%)						
SULT1E1q						
UGT1A1[5] (14%)						
UGT1A1q						
UGT1A3/4[5] (2%)						
UGT1A9[6] (21%)	*UGT1A9*	+ 202.1	-927.9	+1332.1	0.720	No interpretation due to *P* >0.25
UGT1A9q	*UGT1A9q*	- 0.2945	- 0.6150	+ 0.0259	0.070	Enzyme catalyzes biotransformation of E1
UGT1A10[6] (21%)						
UGT1A10q	*UGT1A10q*	+ 0.4743	+ 0.1659	+ 0.7827	0.004	Upregulation by substrate E1

Note:

[1], Significant difference in *E1-S* levels (ADT) in PostMP (lower) and PreMP (higher): *P* <0.001 (6.71x10^{-4}).

[2], Significant difference of *CYP1A1* levels (ADT) in smokers (higher) and non smokers (lower): *P* <0.001 (6.03x10^{-5}).

[3], Correlation of E2 (ADT) and E1-S (ADT): R=0.73, *P*<0.001 (1.41x10^{-7}).

[4], Correlation of *SULT1A1* (ADT) and *SULT1A2* (ADT): R=0.55, *P*<0.001 (1.12x10^{-4}).

[5], Correlation of *UGT1A3/4* (ADT) and *UGT1A1* (ADT): R=0.71, *P*<0.001 ($3.32 \text{x} 10^{-7}$).

[6], Correlation of *UGT1A9* (ADT) and *UGT1A10* (ADT): R=0.55, *P*<0.001 ($1.64 \text{x} 10^{-4}$).

*Because of its lipophilicity E1 distributes preferentially into the oil phase.

Comments:

Because of selection of the exVAR Smoking into the model maybe no selection of the exVAR *CYP1A1* (see footnote[2]).

Because of selection of the exVAR E2 into the model maybe no selection of the exVAR E1-S (see footnote[3]).

E1-S in GLT

CO = 1; n = 42; O/exVAR = 8.4; R^2 = 0.63

**exVAR (% <LOQ)**	**Selected in model**	**Regression Coefficient**	**CI 2.5%**	**CI 97.5%**	***P* Value**	**Interpretation**
Age[1]						
Menopause						
Lobule type	Lob1np	- 0.5284	- 1.0982	- 0.0414	0.07	No (simple) interpretation possible
	Lob1p	- 0.4339	- 1.0294	- 0.1617	0.15	No (simple) interpretation possible, collinearity with periMP and postMP unlikely
Oil%	Oil%	- 0.0320	- 0.0495	- 0.0146	<0.001	Distribution oil/aqueous phase* or less production in adipocytes
BMI						
Smoking						
EAD						
E1	E1	+ 0.0025	+ 0.0018	+ 0.0032	<0.001	Precursor of E1-S
STS						
SULT1A1						
SULT1A2						
SULT1A3/4	*SULT1A3/4*	+ 1.5384	- 0.1733	+ 3.2501	0.08	Enzyme catalyzes formation of E1-S
SULT1E1[1]						

Note:

[1], Correlation of Age and *SULT1E1* (GLT): R=0.51, *P*<0.001 ($4.38 \text{x} 10^{-4}$).

*Because of its hydrophilicity E1-S distributes preferentially into the aqueous phase.

E1-S in ADT

CO = 0; n = 44; O/exVAR = 5.5; $R^2 = 0.67$

**exVAR (% <LOQ)**	**Selected in model**	**Regression Coefficient**	**CI 2.5%**	**CI 97.5%**	***P* Value**	**Interpretation**
Age						
Menopause	PeriMP	- 0.1587	- 0.6492	+ 0.3318	0.52	No interpretation due to *P* >0.25
	PostMP	- 0.6313	- 1.2067	- 0.0559	0.03	Circulating E1-S↓ → diffusion into ADT↓ and/or intra-tissue production of E1-S↓
Lobule type						
Oil%	Oil%	- 0.0197	- 0.0443	+ 0.0048	0.11	Distribution oil/aqueous phase* or less production in adipocytes
BMI						
Smoking						
EAD	EE	- 0.3077	- 0.8935	+ 0.2782	0.29	No interpretation due to *P* >0.25
	ERD	+ 1.3965	+ 0.4639	+ 2.3290	0.005	No (simple) interpretation possible
E1	E1	+ 0.0005	+ 0.0003	+ 0.0007	<0.001	Precursor of E1-S
STS	*STS*	+ 0.0138	+ 0.0045	+ 0.0231	0.005	Upregulation by substrate E1-S
SULT1A1[1]						
SULT1A2[1]	*SULT1A2*	- 3.2271	- 7.4364	+ 0.9821	0.13	Downregulation by product E1-S
SULT1A3/4						
SULT1E1 (35%)						
SULT1E1q						

Note:

[1], Correlation of *SULT1A1* (ADT) and *SULT1A2* (ADT): R=0.55, *P*<0.001 ($1.12x10^{-4}$).

*Because of its hydrophilicity E1-S distributes preferentially into the aqueous phase.

Comment: Because of selection of the exVAR *SULT1A2* into the model, maybe no selection of the exVAR *SULT1A1* (see footnote[1]).

2-MeO-E1 in ADT*

CO = 0; n = 20**; O/exVAR = 6.7; R^2 = 0.42

**exVAR (% <LOQ)**	**Selected in model**	**Regression Coefficient**	**CI 2.5%**	**CI 97.5%**	***P* Value**	**Interpretation**
Age						
Menopause						
Lobule type						
Oil%						
Smoking[1]						
EAD						
E1	E1	+ 0.0003	<0.0001	+ 0.0005	0.03	Precursor of 2-MeO-E1
COMT[2]						
CYP1A1[1]						
CYP1B1[2]						
GUSB						
NQO1						
STS						
SULT1A1	*SULT1A1*	- 28.00	- 64.95	+ 8.94	0.13	Enzyme catalyzes biotransformation of 2-MeO-E1
SULT1E1 (30%)						
SULT1E1q						
UGT1A9 (10%)	*UGT1A9q*	- 0.8593	- 1.7275	+ 0.0089	0.05	Enzyme catalyzes biotransformation of 2-MeO-E1
UGT1A9q						

Note:

[1], Significant difference of *CYP1A1* levels (ADT) in smokers (higher) and non smokers (lower): *P* <0.001 (*6*.03x10^{-5}).

[2], Correlation of *COMT* (ADT) and *CYP1B1* (ADT): R=0.70, *P*<0.001 (0.0005).

*, The model for 2-MeO-E1 in GLT was not calculated because only 13% of samples exhibited levels >LOQ.

** Only samples with levels >LOD were taken into account, making it necessary to reduce possible exVAR. BMI was not considered as exVAR since E1 levels, which might be affected by BMI, we
Only SULT1A1, SULT1E1, and UGT1A9 were included as exVARs due to higher specific activities for substrate 2-MeO-E1 than SULT1A2, SULT1A3/4, UGT1A1, UGT1A3/4, and UGT1A10 (Pem

E2/E1 in GLT

CO = 0; n = 43; O/exVAR = 8.6; R^2 = 0.36

**exVAR (% <LOQ)**	**Selected in model**	**Regression Coefficient**	**CI 2.5%**	**97.5%**	***P* Value**	**Interpretation**
Age[1]						
Menopause						
Lobule type	Lob1np	- 0.5175	- 0.9595	- 0.0756	0.02	In line with positive association with E1 levels
	Lob1p	+ 0.0564	- 0.3971	+ 0.5098	0.80	No interpretation due to *P* value >0.25
Oil%						
IaaGLT						
BMI						
Smoking						
EAD						
CYP1A1 (2%)						
CYP1B1						
CYP19A1	*CYP19A1*	- 59.86	-104.19	-15.54	<0.01	Not associated with E1 and E2 in GLT, no (simple) interpretation possible
GUSB						
HSD17B1						
HSD17B2						
STS						
SULT1A1	*SULT1A1*	+ 38.66	+ 1.77	+ 75.54	0.04	Not associated with E1 and E2 in GLT, no (simple) interpretation possible
SULT1A2						
SULT1A3/4						
SULT1E1[1]						
UGT1A9[2] (16%)	*UGT1A9*	- 1431.4	-2235.9	- 627.9	<0.001	In line with positive association of *UGT1A9*q with E1 levels
UGT1A9q						
UGT1A10[2] (14%)						
UGT1A10q						

Note:

[1], Correlation of Age and *SULT1E1* (GLT): R=0.51, *P*<0.001 ($4.38x10^{-4}$).

[2], Correlation of *UGT1A9* (GLT) and *UGT1A10* (GLT): R=0.69, *P*<0.001 ($8.15x10^{-7}$).

Comment: Because of selection of the exVAR *UGT1A9* into the model, maybe no selection of the exVAR *UGT1A10* (see footnote[2]).

E2/E1 in ADT

CO = 0; n = 44; O/exVAR = 6.3; R^2 = 0.47

**exVAR (% <LOQ)**	**Selected in model**	**Regression Coefficient**	**CI 2.5%**	**CI 97.5%**	***P* Value**	**Interpretation**
Age						
Menopause						
Lobule type	Lob1np	- 0.6747	- 1.0640	- 0.2844	0.001	In line with negative association with E2 levels
	Lob1p	- 0.8664	- 1.2540	- 0.4788	<0.001	In line with negative association with E2 levels
Oil%						
BMI						
Smoking	Smoking	- 0.3931	- 0.7573	- 0.0290	0.04	Not associated with E1 and E2 in ADT, no (simple) interpretation possible
EAD						
CYP1A1 (5%)						
CYP1A1q	*CYP1A1q*	- 0.5051	- 1.2441	+ 0.2339	0.17	Higher impact of negative association of *CYP1A1* with E2 levels, than with E1 levels
CYP1B1						
CYP19A1	*CYP19A1*	- 13.14	-21.93	- 4.35	0.005	In line with positive association with E1 levels and negative association with E2 levels
GUSB						
HSD17B1						
HSD17B2						
STS						
SULT1A1[1]						
SULT1A2[1]						
SULT1A3/4						
SULT1E1 (34%)						
SULT1E1q	*SULT1E1q*	- 0.5142	- 0.8290	- 0.1992	0.002	In line with negative association of *SULT1E1*q with E2 levels
UGT1A1[2] (14%)						
UGT1A1q						
UGT1A3/4[2] (5%)						
UGT1A9[3] (20%)	*UGT1A9*	+ 474.2	-41.81	+990.1	0.07	Not associated with E1 in ADT, no (simple) interpretation possible
UGT1A9q						
UGT1A10[3] (21%)						
UGT1A10q						

Note:

[1], Correlation of *SULT1A1* (ADT) and *SULT1A2* (ADT): R=0.55, *P*<0.001 ($1.12x10^{-4}$).

[2], Correlation of *UGT1A3/4* (ADT) and *UGT1A1* (ADT): R=0.71, *P*<0.001 ($3.32x10^{-7}$).

[3], Correlation of *UGT1A9* (ADT) and *UGT1A10* (ADT): R=0.55, *P*<0.001 ($1.64x10^{-4}$).

Comment: Because of selection of the exVAR *UGT1A9* into the model, maybe no selection of the exVAR *UGT1A10* (see footnote[3]).

E1-S/E1 in GLT

CO=0; n=43; O/exVAR=8.6; R^2=0.47

**exVAR (% <LOQ)**	**Selected in model**	**Regression Coefficient**	**CI 2.5%**	**CI 97.5%**	***P* Value**	**Interpretation**
Age[1]						
Menopause						
Lobule type						
Oil%	Oil%	- 0.0353	- 0.0501	- 0.0205	<0.001	In line with positive association with E1 levels and negative association with E1-S levels
BMI						
Smoking						
EAD						
CYP1A1 (2%)	*CYP1A1*	- 4.0681	- 7.3147	- 0.8214	0.02	Not associated with E1 in GLT, no (simple) interpretation possible
CYP1B1	*CYP1B1*	+ 0.1034	- 0.0534	- 0.2602	0.19	Not associated with E1 in GLT, no (simple) interpretation possible
CYP19A1						
GUSB						
HSD17B1						
HSD17B2						
STS	*STS*	- 0.1201	- 0.2531	- 0.0130	0.08	Not associated with E1 and E1-S in GLT, no (simple) interpretation possible
SULT1A1						
SULT1A2						
SULT1A3/4	*SULT1A3/4*	+ 2.4129	+ 0.5665	+ 4.2594	0.01	In line with positive association with E1-S levels
SULT1E1[1]						
UGT1A9[2] (17%)						
UGT1A9q						
UGT1A10[2] (14%)						
UGT1A10q						

Note:

[1], Correlation of Age and *SULT1E1* (GLT): R=0.51, *P*<0.001 ($4.38x10^{-4}$).

[2], Correlation of *UGT1A9* (GLT) and *UGT1A10* (GLT): R=0.69, *P*<0.001 ($8.15x10^{-7}$).

E1-S/E1 in ADT

CO = 0; n = 44; O/exVAR = 4.0; R^2 = 0.67

exVAR (% <LOQ)	Selected in model	Regression Coefficient	CI 2.5%	CI 97.5%	*P* Value	Interpretation
Age						
Menopause						
Lobule type						
Oil%	Oil%	- 0.0367	- 0.0534	- 0.0200	<0.001	In line with positive association with E1 levels and negative association with E1-S levels
BMI	BMI	- 0.0341	- 0.0851	- 0.0168	0.18	Not associated with E1 and E1-S in ADT, no (simple) interpretation possible
Smoking						
EAD	EE	- 0.4139	- 0.9043	+ 0.0764	0.095	In line with negative association with E1-S levels
	ERD	+ 1.1569	+ 0.5155	+ 1.7983	<0.001	In line with positive association with E1-S levels
CYP1A1 (5%)						
CYP1A1q	*CYP1A1q*	- 1.3722	- 2.2852	- 0.4592	0.004	Not in line with negative association of *CYP1A1* with E1 levels
CYP1B1	*CYP1B1*	+ 0.1001	- 0.0095	+ 0.2098	0.07	Not associated with E1 in ADT, no (simple) interpretation possible
CYP19A1	*CYP19A1*	- 21.2748	-31.0745	- 11.4751	<0.001	In line with positive association with E1 levels
GUSB						
HSD17B1						
HSD17B2						
STS	*STS*	+ 0.0017	- 0.0081	+ 0.0114	0.72	No interpretation due to *P* >0.25
SULT1A1[1]						
SULT1A2[1]						
SULT1A3/4						
SULT1E1 (34%)	*SULT1E1*	+ 284.0	-128.2	-696.1	0.17	Not associated with E1 and E1-S in ADT, no (simple) interpretation possible
SULT1E1q						
UGT1A1[2] (14%)						
UGT1A1q						
UGT1A3/4[2] (5%)						
UGT1A9[3] (20%)						
UGT1A9q	*UGT1A9q*	+ 0.6739	+ 0.2479	+ 1.0999	0.003	Not associated with E1 in ADT, no (simple) interpretation possible
UGT1A10[3] (21%)						
UGT1A10q	*UGT1A10q*	- 0.6918	- 0.2881	- 1.0954	0.001	Not associated with E1 in ADT, no (simple) interpretation possible

Note:

[1], Correlation of SULT1A1 (ADT) and SULT1A2 (ADT): R=0.55, P<0.001 (1.12×10^{-4}).

[2], Correlation of UGT1A3/4 (ADT) and UGT1A1 (ADT): R=0.71, P<0.001 (3.32×10^{-7}).

[3], Correlation of UGT1A9 (ADT) and UGT1A10 (ADT): R=0.55, P<0.001 (1.64×10^{-4}).

ADT/GLT of E2

CO = 0; n = 45; O/exVAR = 7.5; R^2 = 0.38

exVAR (% <LOQ)	Selected in model		Regression Coefficient	CI 2.5%	CI 97.5%	*P* Value	Interpretation
Age							
Menopause	PeriMP	-	0.2038	- 0.5699	+ 0.1624	0.27	No interpretation due to *P* >0.25
	PostMP	-	0.7252	- 1.1182	- 0.3322	<0.001	Not associated with E2 in GLT and ADT, no (simple) interpretation possible
Lobule type							
Oil% ADT	Oil% ADT	+	0.0194	+ 0.0029	+ 0.0360	0.02	Distribution of E2 in oil/aqueous phase or production in adipocytes
Oil% GLT	Oil% GLT	-	0.0163	- 0.0271	- 0.0054	0.004	Distribution of E2 in oil/aqueous phase or production in adipocytes
BMI							
Smoking							
EAD	EE	-	0.5443	- 1.0172	- 0.0714	0.03	Not associated with E2 in GLT and ADT, no (simple) interpretation possible
	ERD	+	0.0363	- 0.6886	+ 0.7611	0.92	No interpretation due to *P* >0.25

ADT/GLT of E1

CO = 0; n = 45; O/exVAR = 9.0; R^2 = 0.67

exVAR (% <LOQ)	Selected in model		Regression Coefficient	CI 2.5%	CI 97.5%	*P* Value	Interpretation
Age							
Menopause	PeriMP	+	0.0154	- 0.2256	+ 0.2565	0.90	No interpretation due to *P* >0.25
	PostMP	+	0.2596	- 0.0098	+ 0.5290	0.06	Higher impact of negative association with E1 levels in GLT, than with E1 levels in ADT
Lobule type							
Oil% ADT	Oil% ADT	+	0.0200	+ 0.0084	+ 0.0315	0.001	In line with positive association with E1 levels in ADT
Oil% GLT	Oil% GLT	-	0.0271	- 0.0346	- 0.0195	<0.001	In line with positive association with E1 levels in GLT
BMI							
Smoking	Smoking	-	0.1906	- 0.4211	- 0.0399	0.10	In line with positive association with E1 levels in GLT
EAD							

Oil% in GLT
CO=0; n=45; O/exVAR=45; R^2=0.26

exVAR (% <LOQ)	Selected in model	Regression Coefficient	CI 2.5%	CI 97.5%	*P* Value	Interpretation
Age						
Menopause[1]						
Lobule type						
IaaGLT	IaaGLT	+ 0.8106	+ 0.4054	+ 1.2157	<0.001	Area covered by adipocytes which contain lipids ↑
BMI						
Smoking						
EE						
E2[1] (7%)						
E2q						

Note:
[1], Significant difference of E2 levels (ADT) in PostMP (lower) and PreMP (higher): *P* <0.001 (7.79x10^{-4}).

Oil% in ADT
CO=2; n=43; O/exVAR=8.6; R^2=0.20

exVAR (% <LOQ)	Selected in model	Regression Coefficient	CI 2.5%	CI 97.5%	*P* Value	Interpretation
Age	Age	+ 0.0032	+ 0.0003	+ 0.0061	0.03	No (simple) interpretation possible
Menopause[1]						
Lobule type	Lob1np	+ 0.0904	+ 0.0085	+ 0.1723	0.03	No (simple) interpretation possible
	Lob1p	+ 0.0365	- 0.0534	+ 0.1264	0.42	No interpretation due to *P* >0.25
BMI	BMI	+ 0.0049	- 0.0023	+ 0.0122	0.18	Storage lipids within adipocytes ↑
Smoking						
EE						
E2[1]	E2	+ 0.0001	<0.0001	+ 0.0002	0.03	Adipogenesis and/or lipogenesis ↑

Note
[1], Significant difference of E2 levels (ADT) in PostMP (lower) and PreMP (higher): *P* <0.001 (7.79x10^{-4}).
Comment: Because of selection of the exVAR E2 into the model, possibly no selection of the exVAR Menopause.

***CYP19A1* in ADT**

CO=0; n=44; O/exVAR=22.0; R^2=0.28

**exVAR (% <LOQ)**	**Selected in model**	**Regression Coefficient**	**CI 2.5%**	**97.5%**	***P* Value**	**Interpretation**
Age						
Menopause						
Lobule type						
BMI						
Smoking						
EAD	EE	- 1.4359	- 2.1020	- 0.7699	<0.001	In line with observations regarding CYP19A1 protein in the endometrium[2]
	ERD	- 0.1175	- 1.2148	+ 0.9798	0.83	No interpretation due to *P* >0.25
AHR						
ARNT[1]						
ESR1						
ESR2[1]						
NR1I2						
NR1I3 (39%)						
*NR1I3*q						

Note:

[1], Correlation of *ARNT* (ADT) and *ESR2* (ADT): R=0.56, P<0.001 (6.68×10^{-5}).

[2], Maia H, Jr., Haddad C, Pinheiro N, Casoy J (2013) The effect of oral contraceptives on aromatase and Cox-2 expression in the endometrium of patients with idiopathic menorrhagia or adenomyosis. Int J Womens Health 5:293-299. https://doi.org/10.2147/IJWH.S45093

GUSB in ADT

CO=3; n=41; O/exVAR=10.3; R^2=0.49

**exVAR (% <LOQ)**	**Selected in model**	**Regression Coefficient**	**CI 2.5%**	**97.5%**	***P* Value**	**Interpretation**
Age						
Menopause						
Lobule type						
BMI	BMI	+ 0.0125	- 0.0021	+ 0.0271	0.09	No (simple) interpretation possible
Smoking						
EAD						
AHR	*AHR*	+ 0.0711	+ 0.0354	+ 0.1068	<0.001	No (simple) interpretation possible
ARNT[1]						
ESR1						
ESR2[1]	*ESR2*	+ 2.0470	+ 0.8731	+ 3.2207	0.001	Possible crosstalk with ESR-mediated signaling via Sp1 and AP-2[2]
NR1I2						
NR1I3 (39%)			- 0.2662			
*NR1I3*q	*NR1I3*q	- 0.1418		- 0.0174	0.03	No (simple) interpretation possible
NFE2L2						

[1], Correlation of *ARNT* (ADT) and *ESR2* (ADT): R=0.56, P<0.001 ($6.68x10^{-5}$).

[2], Naz H, Islam A, Waheed A, Sly WS, Ahmad F, Hassan I (2013) Human beta-glucuronidase: structure, function, and application in enzyme replacement therapy. Rejuvenation Res 16:352-63. https://doi.org/10.1089/rej.2013.1407

Comment: Because of selection of the exVAR *ESR2* into the model, maybe no selection of the exVAR *ARNT* (see footnote[1]).

HSD17B2 **in ADT**

CO=0; n=44; O/exVAR=11.0; R^2=0.37

**exVAR (% <LOQ)**	**Selected in model**	**Regression Coefficient**	**CI 2.5%**	**CI 97.5%**	***P*** **Value**	**Interpretation**
Age						
Menopause	PeriMP	+ 0.3267	- 0.5006	+ 1.1540	0.43	No interpretation due to *P* >0.25
	PostMP	- 0.9725	- 1.8433	- 0.1017	0.03	No (simple) interpretation possible
Lobule type						
BMI	BMI	- 0.1109	- 0.1987	- 0.0231	0.01	No (simple) interpretation possible
Smoking	Smoking	- 0.9946	- 1.8410	- 0.1482	0.02	No (simple) interpretation possible
EAD						
AHR						
ARNT[1]	*ARNT*	- 0.7036	- 1.8410	- 0.1482	<0.001	No (simple) interpretation possible
ESR1						
ESR2[1]	*ESR2*	+ 6.8158	+ 0.4930	+13.1387	0.04	Possible crosstalk with ESR-mediated signaling via Sp1 and Sp3[2]
NR1I2	*NR1I2*	+ 177.7971	+64.1511	+291.4431	0.003	No (simple) interpretation possible
NR1I3 (39%)						
*NR1I3*q						

Note:

[1], Correlation of the levels of *ARNT* (ADT) and *ESR2* (ADT): R=0.56, P<0.001 (6.68x10^{-5}).

[2], Cheng YH, Yin P, Xue Q, Yilmaz B, Dawson MI, Bulun SE (2008) Retinoic acid (RA) regulates 17beta-hydroxysteroid dehydrogenase type 2 expression in endometrium: interaction of RA receptors with specificity protein (SP) 1/SP3 for estradiol metabolism. J Clin Endocrinol Metab 93:1915-1923. https://doi.org/10.1210/jc.2007-1536

Comment: *P* values of the exVARs *ARNT* and *ESR2* are possibly too high (see footnote[1]).
