# Supplementary material for: Influence of breast cancer risk factors and intramammary biotransformation on estrogen homeostasis in the human breast
Source: Arch Toxicol. 2020 Jun 22;94(9):3013–25. doi: 10.1007/s00204-020-02807-1 (PMC7415756; doi:10.1007/s00204-020-02807-1)
Supplement: Supplementary file 5 — Supplementary file5 (PDF 2830 kb) [file 204_2020_2807_MOESM5_ESM.pdf]

Archives of Toxicology

Influence of breast cancer risk factors and intramammary biotransformation on estrogen homeostasis in the human breast

Daniela Pemp, Leo N. Geppert, Claudia Wigmann, Carolin Kleider, René Hauptstein, Katja Schmalbach, Katja Ickstadt, Harald L. Esch, Leane Lehmann*

***Corresponding author:**
Prof. Dr. Leane Lehmann, Chair of Food Chemistry, University of Würzburg, Am Hubland, D-97074 Würzburg, Germany. Phone: +49 931 318-5481. Email: leane.lehmann@uni-wuerzburg.de.

Online Resource 5 Distribution of age of all 47 women (A) as well as frequency of occurrence within a given age category of 19 nulliparous women (B) contributing specimens to the study compared to the general adult female population in Germany (age distribution as of 31st December 2010; frequency of occurrence of nulliparous women in 2016). -, no data available

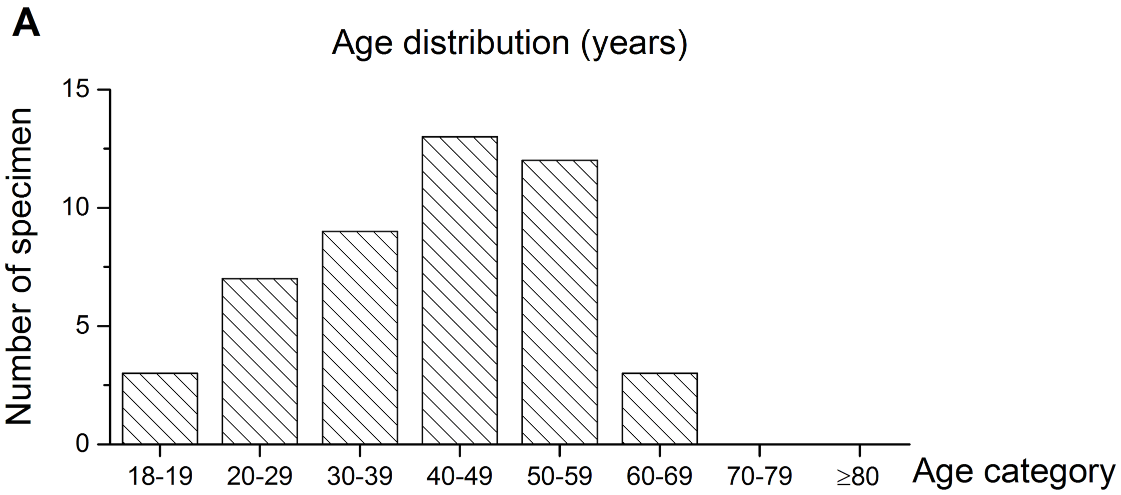

	Percentages in the respective age category								Median (age)
	18-19	20-29	30-39	40-49	50-59	60-69	70-79	≥80	
**study**	6%	15%	19%	28%	26%	6%	0%	0%	44.0
**general**	2%	14%	14%	19%	17%	13%	13%	8%	47.5

B

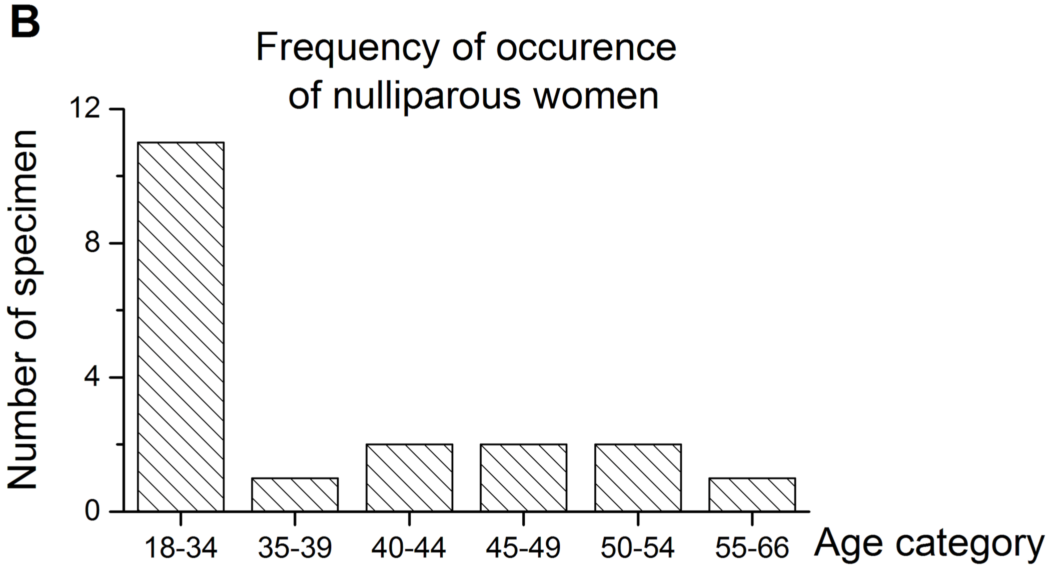

Percentages in the respective age category

	18-34	35-39	40-44	45-49	50-54	55-66
**study**	73%	25%	29%	33%	22%	17%
**general**	-	25%	21%	20%	19%	-

References

Data sources for (A)

Federal Agency for Civic Education, Germany. http://www.bpb.de/nachschlagen/zahlen-und-fakten/soziale-situation-indeutschland/61538/altersgruppen. Accessed 17th January 2020

Federal Ministry of Education and Research, Germany. http://www.datenportal.bmbf.de/portal/de/Tabelle-0.14.html. Accessed 17th January 2020

Federal Institute for Population Research, Germany. https://www.laenderdaten.de/bevoelkerung/medianalter.aspx. Accessed 17th January 2020

Data source for (B)

Statistisches Bundesamt, Germany. https://www.destatis.de/DE/Themen/Gesellschaft-Umwelt/Bevoelkerung/Geburten/Tabellen/kinderlosigkeit.html;jsessionid=DF0FD4A36EDEFD7717CE5A99EFA26454.internet731. Accessed 17th January 2020
