# Supplementary material for: Influence of breast cancer risk factors and intramammary biotransformation on estrogen homeostasis in the human breast
Source: Arch Toxicol. 2020 Jun 22;94(9):3013–25. doi: 10.1007/s00204-020-02807-1 (PMC7415756; doi:10.1007/s00204-020-02807-1)
Supplement: Supplementary file 6 — Supplementary file6 (PDF 1924 kb) [file 204_2020_2807_MOESM6_ESM.pdf]

*Archives of Toxicology*

# Influence of breast cancer risk factors and intramammary biotransformation on estrogen homeostasis in the human breast

Daniela Pemp, Leo N. Geppert, Claudia Wigmann, Carolin Kleider, René Hauptstein, Katja Schmalbach, Katja Ickstadt, Harald L. Esch, Leane Lehmann*

***Corresponding author:**
Prof. Dr. Leane Lehmann, Chair of Food Chemistry, University of Würzburg, Am Hubland, D-97074 Würzburg, Germany. Phone: +49 931 318-5481. Email: leane.lehmann@uni-wuerzburg.de.

**Online Resource 6** Classification of body mass index (BMI) of all 47 women contributing specimens to the present study compared to the general adult female population in Germany (BMI survey period 2008-2011).

BMIs were categorized according to WHO into normal weight (BMI 18.50 - 24.99), pre-obese (BMI 25.00 - 29.99), and obese (BMI ≥30). Data for the BMI of the general female population in Germany were published by Mensink et al. (2013)

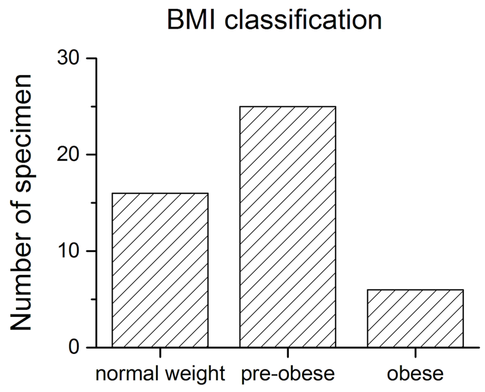


| | Percentages | | | Median (BMI) |
|---|---|---|---|---|
| **study** | 34% | 53% | 13% | 26.1 |
| **general** | 45% | 29% | 24% | 25.4 |

## Reference

Mensink GB, Schienkiewitz A, Haftenberger M, Lampert T, Ziese T, Scheidt-Nave C (2013) [Overweight and obesity in Germany: results of the German Health Interview and Examination Survey for Adults (DEGS1)]. Bundesgesundheitsblatt Gesundheitsforschung Gesundheitsschutz 56:786-794. https://doi.org/10.1007/s00103-012-1656-3
