# Supplementary material for: Influence of breast cancer risk factors and intramammary biotransformation on estrogen homeostasis in the human breast
Source: Arch Toxicol. 2020 Jun 22;94(9):3013–25. doi: 10.1007/s00204-020-02807-1 (PMC7415756; doi:10.1007/s00204-020-02807-1)

Archives of Toxicology

Influence of breast cancer risk factors and intramammary biotransformation on estrogen homeostasis in the human breast

Daniela Pemp, Leo N. Geppert, Claudia Wigmann, Carolin Kleider, René Hauptstein, Katja Schmalbach, Katja Ickstadt, Harald L. Esch, Leane Lehmann*

***Corresponding author:**
Prof. Dr. Leane Lehmann, Chair of Food Chemistry, University of Würzburg, Am Hubland, D-97074 Würzburg, Germany. Phone: +49 931 318-5481. Email: leane.lehmann@uni-wuerzburg.de.

Online Resource 7. Occurrence of breast glandular tissues (GLT, isolated from mixed tissues) exhibiting a significant higher relative area covered by inter- and intrastromal adipocytes (laaGLT, large-adipocyte-area GLT) and of GLT (directly isolated) with a small relative area covered by adipocytes (saaGLT, small-adipocyte-area GLT) in specimen with regard to body mass index (BMI) and lobule type classification (Lob1np, lobule type 1 nulliparous, Lob1p, lobule type 1 parous, Lob2/3, lobule type 2/3).

Contingency analysis of distribution of BMI and lobule type was performed using Chi-Square test for trend and Chi-Square test, respectively. No significant difference in distribution regarding BMI (P = 0.3985) and lobule type (P = 0.2791) was observed between women exhibiting laaGLT and saaGLT.

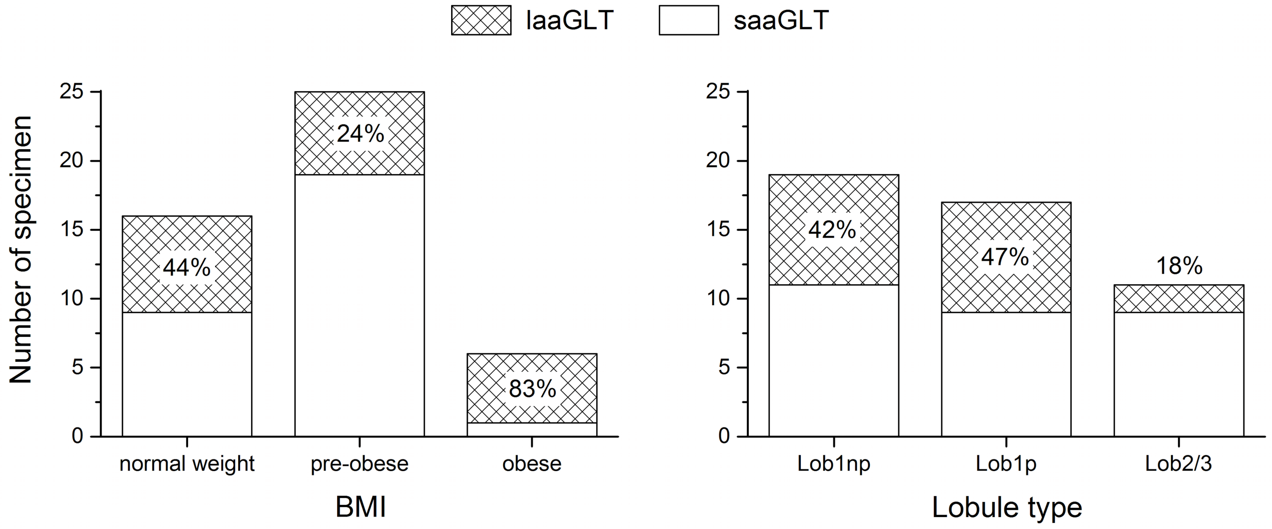

Supplement: Supplementary file 7 — Supplementary file7 (PDF 3152 kb) [file 204_2020_2807_MOESM7_ESM.pdf]
