# Supplementary material for: Influence of breast cancer risk factors and intramammary biotransformation on estrogen homeostasis in the human breast
Source: Arch Toxicol. 2020 Jun 22;94(9):3013–25. doi: 10.1007/s00204-020-02807-1 (PMC7415756; doi:10.1007/s00204-020-02807-1)

Archives of Toxicology

Influence of breast cancer risk factors and intramammary biotransformation on estrogen homeostasis in the human breast

Daniela Pemp, Leo N. Geppert, Claudia Wigmann, Carolin Kleider, René Hauptstein, Katja Schmalbach, Katja Ickstadt, Harald L. Esch, Leane Lehmann*

***Corresponding author:**
Prof. Dr. Leane Lehmann, Chair of Food Chemistry, University of Würzburg, Am Hubland, D-97074 Würzburg, Germany. Phone: +49 931 318-5481. Email: leane.lehmann@uni-wuerzburg.de.

Online Resource 8A. Mosaic plots characterizing the 43 breast glandular tissues (GLT) / 44 breast adipose tissues (ADT) contributing data to linear regression models with respect to 4 categorical explanatory variables tested as well as to body mass index (BMI), which was tested as continuous explanatory variable.

To facilitate reading of mosaic plots, colors differentiate categories of menopausal (MP) status or BMI of the top axis, boxes with/without pattern indicate smokers/non-smokers (right axis) and dark/light colors indicate intake of estrogen-active drugs (EAD) yes/no (bottom axis). Numbers inside boxes represent numbers of specimens exhibiting the respective characteristics.

*, minus 1 specimen for GLT

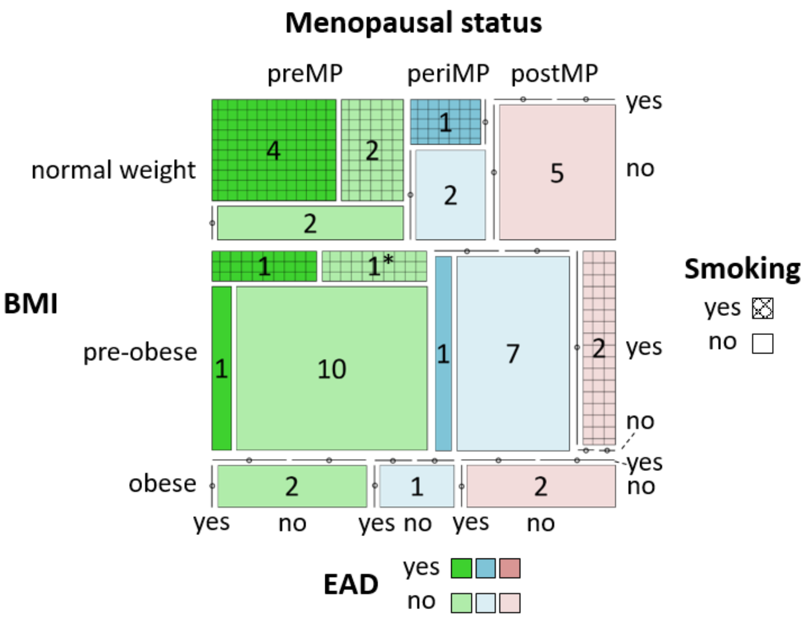
Menopausal status
preMP periMP postMP
BMI
normal weight
pre-obese
obese
Smoking
yes
no
EAD
yes
no

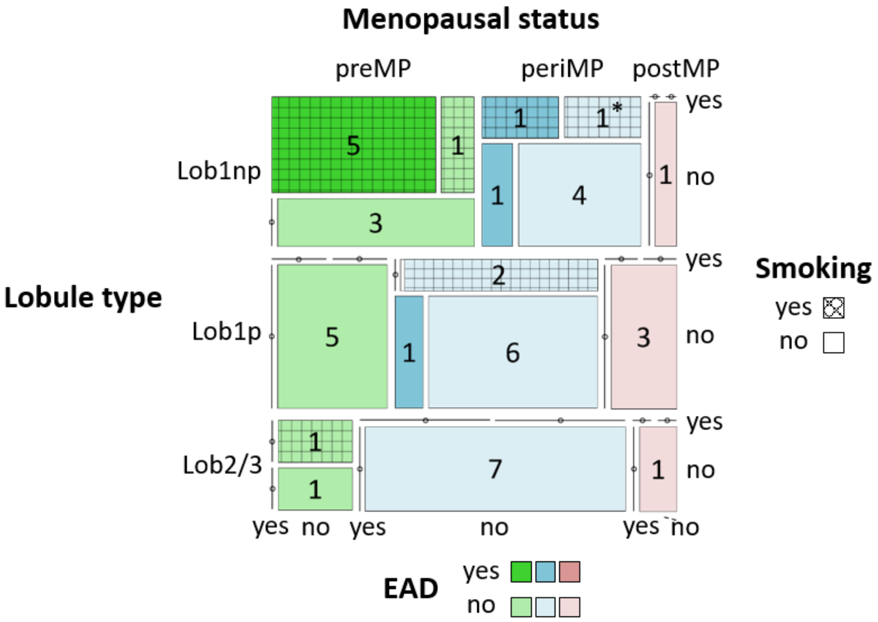
Menopausal status
preMP periMP postMP
Lobule type
Lob1np
Lob1p
Lob2/3
Smoking
yes
no
EAD
yes
no

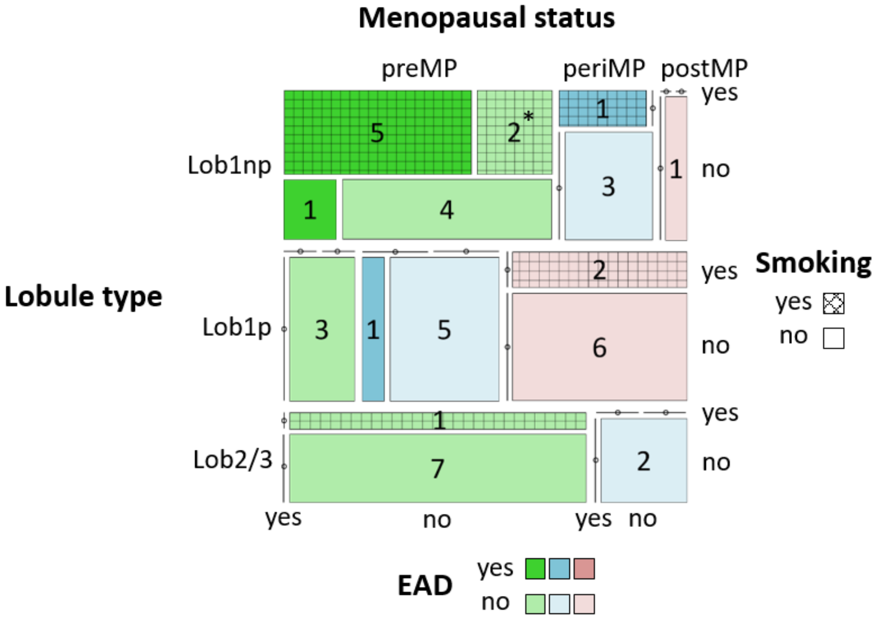
Menopausal status
preMP periMP postMP
Lobule type
Lob1np
Lob1p
Lob2/3
Smoking
yes
no
EAD
yes
no

Online Resource 8B. Mosaic plot characterizing the 45 breast glandular tissue (GLT) specimens contributing data to the linear regression model describing mass percentage of oil.

Eighteen GLT were isolated from mixed tissues (i.e. GLT exhibiting larger area of adipocytes, laaGLT yes) and 27 GLT specimens could be isolated directly (laaGLT no). The study population was further classified regarding BMI as well as parity and lobule type of the GLT (lobule type 1 nulliparous, Lob1np, lobule type 1 parous, Lob1p, and lobule type 2/3, Lob2/3).

Numbers in boxes represent numbers of specimens exhibiting the respective characteristics.

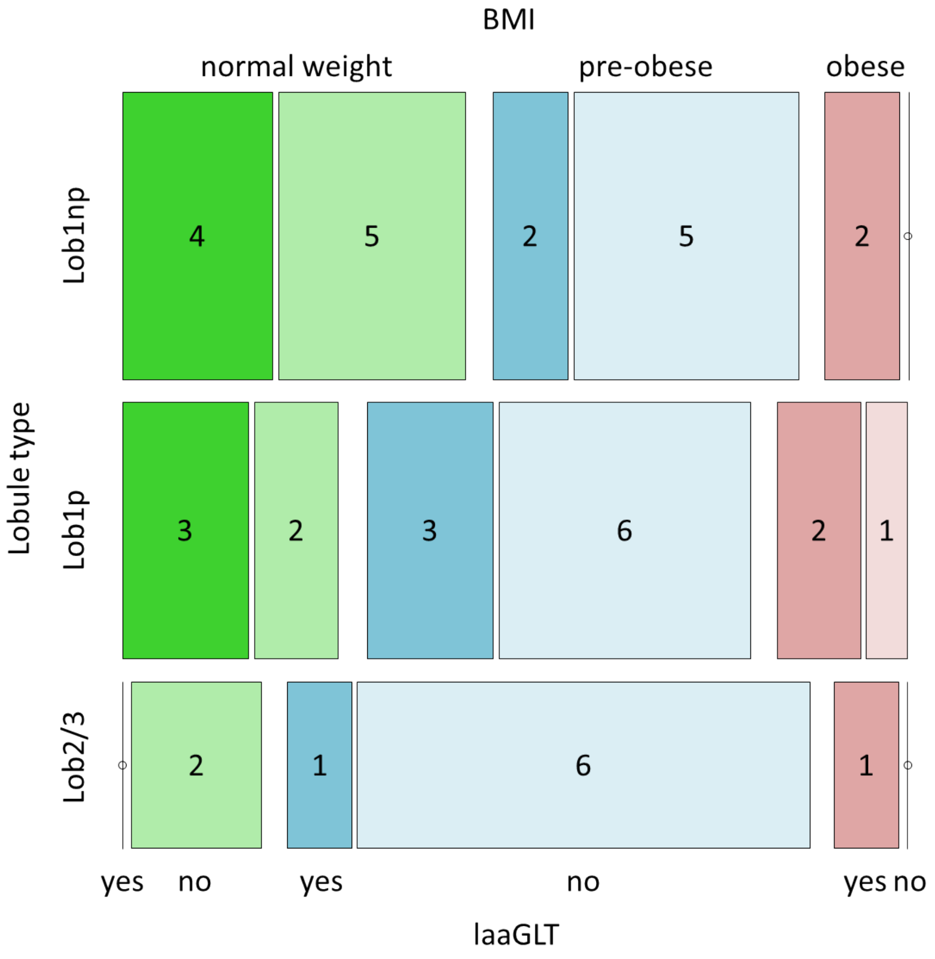

Supplement: Supplementary file 8 — Supplementary file8 (PDF 401 kb) [file 204_2020_2807_MOESM8_ESM.pdf]
